# Inhibition of miR-4763-3p expression activates the PI3K/mTOR/Bcl2 autophagy signaling pathway to ameliorate cognitive decline

**DOI:** 10.7150/ijbs.103225

**Published:** 2024-11-04

**Authors:** Wenxin Qi, Yiwei Ying, Peiru Wu, Naijun Dong, Wenjun Fu, Qian Liu, Natalie Ward, Xin Dong, Robert Chunhua Zhao, Jiao Wang

**Affiliations:** 1 School of Life Sciences, Shanghai University, Shanghai, China.; 2 School of Medicine, Shanghai University, Shanghai, China.; 3 Jinan Microecological Biomedicine Shandong Laboratory, Jinan 250021, China.; 4 Banner Ocotillo Medical Center, 1405 S Alma School Rd, Chandler, AZ 85286, USA.; 5 Centre of Excellence in Tissue Engineering, Chinese Academy of Medical Sciences, Beijing, China.; 6 Beijing Key Laboratory of New Drug Development and Clinical Trial of Stem Cell Therapy (BZ0381), Beijing, China.; 7 Institute of Basic Medical Sciences, Chinese Academy of Medical Sciences, School of Basic Medicine, Peking Union Medical College, Beijing, China.

**Keywords:** AD-MCI, apoptosis, autophagy, phosphatidylserine

## Abstract

Cognitive decline and memory impairment are subsequently result in neuronal apoptosis and synaptic damage. Aberrant regulation of microRNAs has been implicated in the pathogenesis of Alzheimer's disease (AD) and may play a pivotal role in the early stages of the disease. In this study, we identified the critical role of miR-4763-3p in AD pathogenesis, focusing on early-stage mild cognitive impairment (AD-MCI). Leveraging fluorescence *in situ* hybridization, we observed miR-4763-3p upregulation in AD hippocampal tissue, colocalizing with Aβ and Tau. Antagomir-mediated inhibition of miR-4763-3p ameliorated cognitive decline in AD-MCI mice. RNA-seq and functional assays revealed that miR-4763-3p targets ATP11A, and antagomir enhancing inward flipping of the "eat me" phosphatidylserine signal on the surface of neuronal cells, autophagy, and clearance of Aβ/lipofuscin, while reducing neuroinflammation and neuronal apoptosis. Mechanistically, miR-4763-3p modulates the PI3K/AKT/mTOR/Bcl2 pathway, thereby promoting neuronal autophagy and reducing apoptotic crosstalk. These findings underscore miR-4763-3p as a therapeutic target for AD-MCI, offering a novel strategy to enhance neuronal autophagy, alleviate inflammation, and improve cognitive function.

## Introduction

Cognitive decline and memory impairment are commonly observed in brain injury, aging, schizophrenia, and neurodegenerative diseases and have a significant impact on patients' quality of life. Alzheimer's disease (AD) is the most prevalent progressive neurodegenerative disease. According to an epidemiological data project, by 2050, the number of AD patients in the United States will double in comparison with that recorded in 2021 1. AD is rapidly becoming one of the costliest, deadliest and most burdensome diseases of this century 2. The AD brain exhibits a variety of pathological features, including the deposition of senile plaques and neuronal death 3, 4. The downregulation of excitatory and inhibitory genes in the human frontal cortex is one of the most significant changes observed in AD 5. There is currently no effective drug for the treatment of AD, and the irreversibility of this disease necessitates early intervention 6. Accordingly, it is imperative to understand the pathological mechanisms underlying AD and develop targeted drugs.

MicroRNAs (miRNAs) are a class of noncoding RNA molecules, typically consisting of approximately 22 nucleotides, that play a crucial role in post-transcriptional gene regulation and have been implicated in dysregulated gene expression 7-9. Recent studies have identified miR-331-3p and miR-9-5p have been shown to promote autophagy, improve cognitive ability in mice, and serve as diagnostic markers for early- and late-stage AD 10. Overexpression of miR-455-3p can improve synaptic and cognitive function and extend lifespan in mouse models of AD 11. In recent years, research on miRNAs as biomarkers of AD and the manner in which these molecules regulate related signaling pathways has become popular.

The symptoms of AD are marked by an accumulation of protein aggregates (e.g., Aβ and tau) in the brain, which results in an inflammatory environment. This inflammatory environment contributes to the impairment of the autophagy-lysosome system. In turn, damage to this system leads to increased protein accumulation and inflammatory phenotypes, contributing to disease progression 12. Autophagy plays a significant role in the maintenance of organismal homeostasis 13 and closely associated with the development of neurodegenerative diseases such as Alzheimer's disease 14. In neurons, autophagy has a protective effect and is essential for the maintenance and survival of neurons 15. Therefore, targeting and regulating the inflammatory microenvironment in the brain and the inflammatory environment of nerve cells, improving autophagy, and reducing nerve cell damage has become an effective strategy for the treatment of AD.

In this study, upon screening through clinical databases and collected human serum samples, we have discovered that miR-4763-3p is upregulated in the early stages of AD and is positively correlated with age. MiR-4763-3p was significantly upregulated in neurons in the AD brain and colocalized with the Aβ and Tau proteins. Previous research 16 has shown that miR-4763-3p is closely related to cognition. Hence, we delved into the mitigating influence of miR-4763-3p on cognitive deterioration in a preclinical AD mouse model. Specifically, administering miR-4763-3p antagomir demonstrated amelioration of learning and memory impairments, accompanied by reduced neuroinflammation, neuronal loss, and synaptic alterations. Mechanistically, miR-4763-3p forms a feedforward loop with ATP11A and the transcription factor YY1. In the inflammatory environment of the AD brain, inhibiting miR-4763-3p markedly enhances ATP11A expression, mediating inward flipping of phosphatidylserine (PS), thereby reducing neuronal apoptosis and alleviating extracellular inflammation. Furthermore, this feedforward loop improves intracellular homeostasis by enhancing neuronal autophagy, with autophagy and apoptosis being balanced through the PI3K/AKT/mTOR signaling pathway. Our data suggest that miR-4763-3p may serve as a potential therapeutic target for the clinical treatment of AD, providing novel strategies for the treatment of AD.

## Materials and Methods

### Animals

C57BL/6 wild-type (WT) and 3xTg (a transgene with mutated human APP K670N/M671L and MAPT P301L and a knock-in mutation Psen1M146V) mice were obtained from Shanghai Model Organisms Center, Inc. 17. Mice were raised at a constant temperature (22 ± 1°C) with a light/dark cycle of 12/12 h and were provided with free access to food and water. All animals were treated in accordance with international animal research guidelines. The study design was approved by the Animal Ethics Committee of Shanghai University.

### Identification of DEGs and functional annotation

To explore the miRNAs affecting AD, we obtained raw RNA-Seq data downloaded from the GEO database (GEO accession number: GSE120584). The thresholds for the screening of differentially expressed genes (DEGs) were set to |log2FC|>1.5 and P < 0.05. To further explore the potential molecular mechanisms and downstream signaling pathways involved in miR-4763-3p regulation, we conducted sequencing analysis on the hippocampal region of AD-MCI model mice (each group n = 3) that received stereotactic injection of the miR-4763-3p antagomir or NC. To identify DEGs between AD patients and healthy controls, we obtained previously reported raw RNA-seq data from the human postmortem hippocampus (GEO accession number: GSE173955). The thresholds for the screening of DEGs were set to |log2FC| > 1.5 and P < 0.05. The P value was obtained using one-way ANOVA; P < 0.05 was considered to indicate a significant difference between values. DAVID 6.8 was used for functional enrichment analysis of AD-related genes 18.

### Behavioral analysis

#### Novel object recognition test

The novel object recognition (commonly known as NOR) test was performed in an open-field box (40 cm × 40 cm). On the first day, the mice were habitually trained (5 min) and allowed to adapt to the test room for 48 h in an open area. On the third day, two identical objects were presented in opposite quadrants, and the mouse was allowed to freely explore for 5 min. Subsequently, the object was replaced with a new object, and a second 5 min of free exploration was conducted. Noldus software was used to analyze the total movement distance, residence time, number of entries, and average speed at which the mice entered the area around the two objects. Between tests, the instrument was cleaned with 75% ethanol.

#### Morris water maze

Following stereotactic injection, the mice were subjected to the Morris water maze (MWM) test to evaluate their cognitive abilities and memory performance. All mice underwent memory training four times a day, with an interval of 20 min, for four consecutive days. On the fifth day, the mice underwent a memory probe test to evaluate the retention of memory to find the hidden platform. The animals were video contemporaneously recorded via SMART 3.0 software (Panlab HARVARD, MA, USA), and behavioral metrics, including percentage of time in each quadrant, distance traveled, latency, and percentage of distance in each quadrant, were calculated.

#### Y-maze

On the testing day, the mice (6 months old) were allowed to freely explore two arms of the Y-maze for 5 min, while the other arms were blocked. After habituation training, the mice were allowed to freely explore the three arms for 5 min. During exploration, the duration and frequency of entry into the novel open arm were recorded. Spontaneous alternation was defined as consecutive entry into three different arms on the overlapping triplet. The apparatus was cleaned with 75% ethanol between tests.

#### Barnes maze

The Barnes maze test was used to evaluate the spatial memory ability of the mice. Briefly, the Barnes platform is circular, 92 cm in diameter, and contains 20 holes, 19 of which are blocked, leaving only one escape hole. Mice were acclimated to the experimental room one day in advance. During the training phase, each mouse was habituated to the escape hole for 4 min and then to the starting box for 30 s. The mice were then allowed to freely explore the platform, and spatial cues, bright light, and white noise were used to motivate the mice to find the escape hole within 4 min. Any mice that failed to find the escape hole were guided to it and allowed to habituate for 1 min. After three days of training, a 4-min platform probe test was performed, and the time taken and distance traveled by each mouse to enter the escape hole were recorded. Data analysis was performed using SMART software.

### RT-qPCR

The mRNA levels were detected by RT-qPCR. The final reaction contained 2 µg of total RNA, 4 μL of 5× RT Master Mix (ABclonal; Wuhan, China), and RNase-free water (up to 20 μL). cDNA was reverse-transcribed using the following PCR parameters: 55°C for 15 min and 85°C for 5 min. Subsequently, qPCR SYBR Green Master Mix (ABclonal; Wuhan, China) was used for the qPCR amplification reactions. Each reaction contained 1 μL of cDNA sample (100-200 ng/μL), 10 μL of qPCR SYBR Green Master Mix, 0.8 μL (10 µM) of the designated primers, and RNase-free water (up to 20 μL). The qPCR conditions were as follows: 95°C for 3 min, 40 thermal cycles at 95°C for 5 s, and 60°C for 30 s. mRNA levels were normalized to those of *GAPDH,* and the relative gene expression was quantified using the ΔΔCt method. The primers used in this study are detailed in Table S1-3.

### Western blotting

The cells were washed with cold phosphate buffer saline (PBS) and lysed on ice with tissue protein extraction reagent (Beyotime, China) for 30 min, ice bath ultrasound for 1 min, ultrasound for 2 s, with a 10 s interval (SCIENTZ, JY92-IIN). Each homogenate was centrifuged at 12,000 rpm for 30 min at 4°C, the supernatant was collected, and the soluble protein concentration was determined using a BCA-100 protein detection kit (KTD3001, Abbkine, Wuhan, China). A total of 20 μg of medium-quality protein per sample was boiled for 10 min in 5× loading buffer, subjected to sodium dodecyl sulfate-polyacrylamide gel electrophoresis (SDS-PAGE) (Shandong Sparkjade Biotechnology Co., Ltd.), and then transferred to a nitrocellulose membrane. The membranes were blocked with 5% skim milk at room temperature for 2 h, incubated overnight at 4°C with primary antibodies (ABclonal, Wuhan, China), and then incubated with a secondary antibody at room temperature for 1 h. The protein bands were visualized using an Odyssey scanner and associated software (LI-COR Biosciences, USA). Relative protein levels were normalized to those of GAPDH, β-Tubulin (Bioss, China), beta-actin or vinculin.

### Stereotactic injection

3xTg mice were obtained from Shanghai Model Organisms Center, Inc. The mice were divided into five groups (n = 10 per group). Targeted injection into the hippocampus was performed with the miR-4763-3p agomir, miR-4763-3p antagomir, and negative controls (NC: sequence-scrambled miRNA; Table S4, 5). The miR-4763-3p agomir and antagomir and the scrambled control were purchased from RiboBio (Guangzhou, China). The treatment groups were as follows: AD-MCI+miR-4763-3p antagomir NC, AD-MCI+miR-4763-3p agomir NC, AD-MCI+miR-4763-3p antagomir, and AD-MCI+miR-4763-3p agomir. First, we made a longitudinal incision to expose the bregma and set this point to zero. From this point, we determined the hippocampal CA1 area (anterior and posterior: + 2.0 mm, inner and outer ± 0.3 mm, dorsoventral + 1.9 mm). Each mouse was injected with 2 μL of treatment solution within 10 min; the needle was then held for 10 min, after which the needle was slowly retracted. Seven days after stereotaxic injection, the mice were euthanized, and their hippocampi were isolated for total RNA extraction to test the effect of stereotaxic injection. After 7 days of free access to food and water, the behavioral test was conducted.

### Dual-luciferase reporter gene detection

The relationship between hsa-miR-4763-3p and *ATP11A* was verified using a dual luciferase reporter assay kit (Vazyme, China). The binding sequences of miR-4763-3p and *ATP11A* were predicted by RNAhybrid software, and the free energy of their targeted binding sites was analyzed. We designed the mutated (MUT) binding site using the complementary sequence of wild-type (WT) *ATP11A* and constructed the pGL3 Basic reporter plasmid. HEK-293T cells were transfected with ATP11A-MUT (mutant 3'UTR) or ATP11A-WT (luciferase reporter plasmid with the correct sequence) and the miR-4763-3p mimic or NC (Shanghai Ribo, China) for 48 h. After cell collection and lysis, the relative luciferase activity was calculated as the ratio of firefly luciferase to renilla luciferase. Three independent experiments were performed.

### Immunofluorescence imaging

The NeuN, Iba1, Aβ, Tau, ATP11A, Annexin V, SQSMT1 and Caspase 3 levels were monitored by immunostaining (the relevant antibody information is provided in Table S6). Brain tissue slices (n=3) on coverslips were washed three times with 1× PBS for 3 minutes each and fixed in 4% paraformaldehyde (PFA) for 15 minutes. After washing three times with 1× PBS for 3 minutes each, the slices were blocked and permeabilized in 1× PBS containing 1% goat serum and 0.25% Triton™ X-100 and then stained with a primary antibody overnight at 4°C. The following day, the slices were washed three times with 1× PBS for 3 minutes each, incubated with a secondary antibody at room temperature for 1 hour, and washed again. DAPI (Invitrogen, NBP2311561) was used to counterstain the nuclei, which were then incubated in the dark for 5 minutes. Finally, the slices were washed four times with PBST for 5 minutes each to remove excess DAPI and then mounted with mounting solution containing a fluorescence quencher. Images of immunostained brain tissue slices were obtained using confocal fluorescence microscopy (Zeiss). After adjusting the threshold, the fluorescence intensity was quantified using ImageJ® version 1.6.0 software (National Institute of Mental Health/NIH Research Services Branch, Bethesda, Maryland).

### Immunocytochemistry (IHC)

Brain slices (10 μm thick) were obtained with a cryostat and rinsed with 1× PBS three times. Then, these slices were treated with 3% hydrogen peroxide for 30 min to block endogenous peroxidase activity. After incubation with 0.1% Triton X-100 for 20 min to permeabilize the membrane, the slices were blocked with 5% bovine serum albumin (BSA) for 30 min. The brain slices were incubated with the primary antibodies IL-6 and TNF-α overnight at 4°C. After washing with PBST, the slices were probed with a biotinylated secondary antibody and streptomycin-labeled peroxidase solution for 1 h at room temperature and then stained with 3,3'-diaminobenzidine (DAB) reagent for 1 to 10 min at 37°C. After washing, the brain slices were dehydrated with different concentrations of alcohol (75, 80, 95, and 100%), rendered transparent in xylene, and sealed on glass slides. The digital images of all slices were captured using a Pannoramic MIDl.

### Fluorescence *in situ* hybridization (FISH)

A fluorescently labeled miR-4763-3p FISH probe was designed and synthesized by Servicebio (Wuhan, China). Fluorescence-labeled single-strand probes were hybridized. FISH was carried out according to the manufacturer's instructions for SweAMI-FISH (Servicebio). All fluorescence images were captured using a confocal laser microscope (Zeiss, Germany). The miR-4763-3p probe sequence used was CCCGCCCAGCACCAGCCCCTGCCT.

### Flow cytometry

Cells were collected at 48 h post-transfection and plated in a 6-well plate (703001, NEST Biotechnology Co.Ltd). An apoptosis detection kit (CA1020, Bei jing Solarbio Science & Technology Co., Ltd.) was used to measure the levels of apoptosis in the transfected cells. The cells were suspended in 50 μL of 1× binding buffer, to which 5 μL of Annexin V-FITC or 5 μL of anti-PI-PE was added, and the mixture was incubated for 15 minutes at room temperature. Next, 400 μL of 1× binding buffer was added to each sample. Flow cytometry (Beckman Coulter Cytoflex) was used to collect the data.

### ELISA

Tissue: Frozen hippocampal tissue was rapidly homogenized using a homogenizer in PBS containing a protease inhibitor and then centrifuged at 5,000 × g for 10 min to collect the supernatant. The levels of the cytokines IL-6 and TNF-α in the serum and tissue were measured by ELISA kits (JL20268, JL10484, Jianglai biology, Shanghai).

SH-SY5Y cells were plated in 6-well plates at 1 × 10^6^/well and transfected with NC (5 μM), siATP11A (5 μM), OE ATP11A (4 μg of pcDNA3.0-ATP11A-overexpressing plasmid) or vector (4 μg) for 48 h. Supernatants were collected for detection of TNF-α and IL-6 by ELISA.

### Statistical analysis

The data were analyzed and are presented as the mean ± standard error of the mean (SEM) of at least three independent experiments. The analytical methods used were GraphPad Prism version 8 (GraphPad Software, Boston). For comparisons between two groups, a two-tailed unpaired Student's t test was used for normally distributed data. For comparisons involving multiple groups, one-way ANOVA and two-way ANOVA followed by Tukey's multiple comparison test were used for normally distributed datasets. *P* < 0.05 was considered to indicate statistical significance.

## Results

### MiR-4763-3p antagomir ameliorates cognitive decline

**Figure 1:**
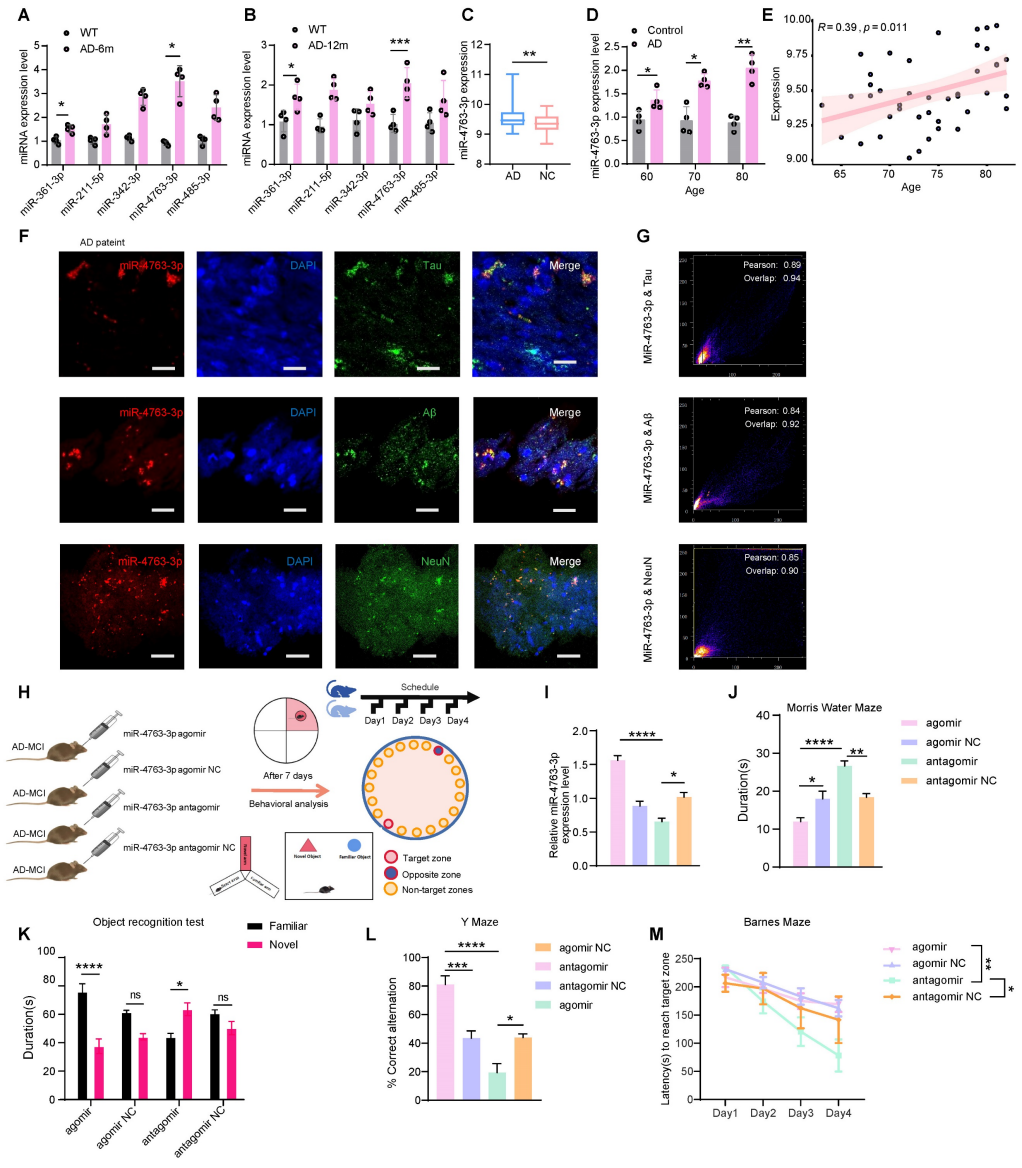
** MiR-4763-3p was highly expressed in the serum and hippocampal tissue of AD patients.** A, B: Expression levels of miRNAs in hippocampal tissues of AD-MCI and WT mice at 6 and 12 months. C: Standardized relative expression of miR-4763-3p in serum samples from AD patients and healthy controls in the GSE120584 dataset. D: miRNA-qPCR assay of clinical serum samples from AD patients (AD) and normal controls (control). (AD: n=30, Control: n=30). E: Correlation analysis of miR-4763-3p levels in APOE4 high-risk AD patients according to patient age. Correlation analysis was performed using the R Studio (version 4.3) cor function and Pearson correlation analysis method. F: Fluorescence *in situ* hybridization (FISH) and immunofluorescence (IF) staining of NeuN, Aβ, tau (green), and miR-4763-3p (red) in AD (Alzheimer's disease patient) brain hippocampal tissue. Nuclei were stained with DAPI (blue). Scale bar, 50 μm. G: Pearson correlation coefficient was used to analyze the co-localization relationship between Mir-4773-3p and NeuN, Aβ and tau. H: Stereotaxic injection of miR-4763-3p antagomir antagomir NC, agomir, agomir NC into the brain of AD-MCI mice and its behavioral detection diagram. I: Expression levels of miR-4763-3p in mouse hippocampal tissue seven days after injection of the miR-4763-3p agomir, agomir NC, antagomir or antagomir NC. J: The Morris water maze test was performed to analyze long-term memory. The duration of occupancy of the platform area was measured (n =10 per group). K: The object recognition test was conducted to assess spatial memory capacity. The duration of exploration of novel objects was recorded (n = 10 per group). L: The Y maze test was conducted to evaluate spatial memory capacity. Percentage of spontaneous alternations during the Y-maze test. (n = 10 per group). M: The Barnes maze test was performed to assess changes in cognitive ability. The latency to reach the target hole was recorded (n = 10 per group). The data are expressed as the mean ± SEM of three independent experiments. *P < 0.05; **P < 0.01; ***P < 0.001; ****P < 0.0001.

To determine the differences in miRNAs between patients with AD and healthy people, we analyzed the noncoding RNA profile data from the serum samples of 50 AD patients and 50 healthy individuals (GSE120584), 424 differentially expressed miRNAs between the AD group and the healthy group were screened (Figure S1A). According to the study, only 5 miRNAs are thought to be cognitively relevant 16, 19-22. MiRNA-qPCR experiments in the hippocampal tissues of AD and WT mice at 6 and 12 months revealed that miR-361-3p and miR-4763-3p exhibited increased expression levels during the early stages of AD and may be accelerate the progression of the disease in the later stages (Figure 1A, B). Therefore this study was to further explore the effects of miR-4763-3p on AD neurons and its impact on cognitive function.

For an in-depth analysis of the relationship with age and risk factors of the sequencing data (GSE120584), the results revealed significant upregulation of miR-4763-3p in the serum of AD patients (Figure 1C) and clinical sample analysis verified that miR-4763-3p expression increased with age in AD patients (Figure 1D). Apolipoprotein E epsilon 4 (APOE4), which is strongly associated with the risk of age-related cognitive decline in individuals without dementia, the strongest genetic risk factor for AD 23, 24. Correlation analysis between the levels of miR-4763-3p and patient age in APOE4 high-risk AD patients revealed a positive association between miR-4763-3p and age (Figure 1E), suggesting that both could be markers for AD detection. Next, we delve deeper into the localization of miR-4763-3p within the brain to investigate its expression and spatial relationship with pathological hallmarks of AD, which may be linked to the function of this miRNA. Fluorescence *in situ* hybridization (FISH) coupled with pearson correlation coefficient analysis of postmortem hippocampal tissue from AD patients revealed a positive correlation between miR-4763-3p expression and levels of Aβ or Tau proteins, accompanied by partial co-localization (Figure 1F, G). MiR-4763-3p also co-localized with neurons (Figure 1 F, G), indicating a potential role for miR-4763-3p in modulating intracellular neuronal mechanisms underlying AD pathogenesis.

To further explore the mechanism of miR-4763-3p in the pathogenesis of AD and its cognitive rescue effects, we stereotactically administered agomir, agomir NC, antagomir, or antagomir NC into the hippocampal CA1 region of AD-MCI mice (Figure 1H). The detection of miR-4763-3p expression in the hippocampal region of the mouse brain showed that demonstrating effectiveness seven days after administration (Figure 1I). Then, we assessed the learning and memory abilities of these mice. The results of the Morris water maze experiment demonstrated that the miR-4763-3p antagomir treated AD-MCI had longer duration of stay and number of platform crossings, while miR-4763-3p agomir group, which spent the shortest amount of time in the target quadrant (Figure 1J, Figure S1B). The results of the novel object recognition test (assessing spatial learning) showed that the AD-MCI-miR-4763-3p agomir group had a lower frequency and duration of exploring objects than did the agomir NC group, and antagomir group spent almost twice as much time exploring novel objects than familiar objects and discovered novel objects more frequently (Figure 1K, Figure S1C, F). The results of the Y-maze experiment showed miR-4763-3p antagomir treatment group alternated at higher levels than the chance level of 50% and had a greater frequency and duration of stay in the novel arm than in the familiar arm, demonstrating their greater spatial learning ability in comparison with other mice (Figure 1L, Figure S1D). In the Barnes maze experiment, AD-MCI mice in the miR-4763-3p antagomir treatment group displayed a significantly shorter latency to reach the target hole during the search stage than did those in the NC and agomir treatment groups, indicating a significant improvement in learning ability and spatial memory following miR-4763-3p antagomir treatment (Figure 1M, Figure S1E). The present findings underscore the potential diagnostic utility and promising therapeutic efficacy of miR-4763-3p antagomir intervention in the context of early-stage AD.

### The miR-4763-3p antagomir rescued neuronal loss and synaptic morphology

**Figure 2:**
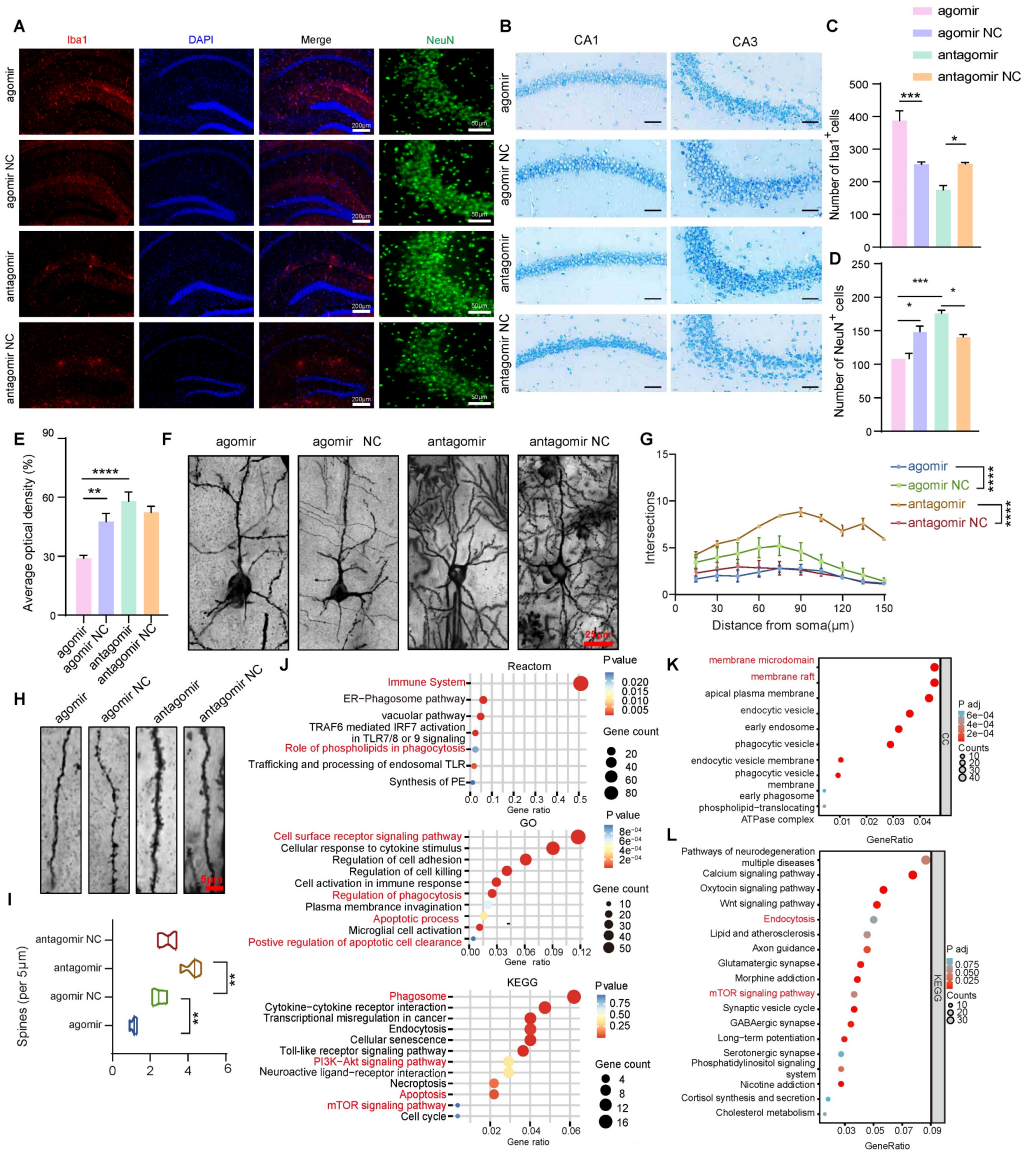
** Neuronal deficits and changes in synaptic morphology in the brains of AD mice were restored after miR-4763-3p antagomir treatment.** A: IF staining of NeuN (green) and Iba1 (red) in mouse brain tissues from the NC antagomir, NC antagomir, NC agomir, and agomir groups. Scale bar, 200 or 50 μm. B: Nissl staining of the CA1 and CA3 regions of the hippocampus from different groups. Scale bar, 50 μm. C-E: Statistical analysis of Iba1^+^ (C) and NeuN^+^ cell numbers (D) and Nissl bodies (E). F: Golgi staining showing dendritic spine morphology in mice injected with the NC antagomir or antagomir. Scale bar, 25 μm. G: Sholl analysis was performed to evaluate the dendritic complexity of the agomir-, agomir NC-, antagomir-, and antagomir NC-injected mice. H: An example of Golgi staining examining the dendritic spine morphology of tertiary neurons from mice injected with the antagomir NC or antagomir. Scale bar, 5 μm. I: Quantitative analysis of spine density (per 5 μm). J: Reactome enrichment analysis, Kyoto Encyclopedia of Genes and Genomes (KEGG) enrichment analysis and Gene Ontology (GO) enrichment analysis of all DEGs. K: GO enrichment analysis of unregulated genes. L: Kyoto Encyclopedia of Genes and Genomes (KEGG) enrichment analysis. Data from GEO database (GSE173955). The data are expressed as the mean ± SEM of three independent experiments. *P < 0.05; **P < 0.01; ***P < 0.001; ****P < 0.0001.

To further investigate the specific mechanisms underlying the improvements in cognitive and memory abilities following miR-4763-3p antagomir treatment, immunofluorescence staining was performed in the hippocampus of AD-MCI mice after stereotactic injection. The experimental results indicate that the number of Iba1-positive cells in the agomir group is significantly higher than that in the other groups, whereas the count of NeuN-positive neuronal cells is the lowest. In comparison to the NC group, the expression of Iba1-positive cells is relatively lower in the antagomir group, and the CA3 brain region exhibits an abundance of NeuN-positive cells. This demonstrates significant neuronal loss and microglial cell proliferation in the miR-4763-3p agomir group when compared to the NC groups. Conversely, following treatment with the miR-4763-3p antagomir, a significant increase of neurons and decreased microglial cells were observed (Figure 2A, C and D). Nissl bodies can serve as a marker of neuronal functional status since these structures decrease or disappear following neuronal damage 25. Interestingly, the staining intensity of Nissl bodies in the CA2 and CA3 hippocampal regions was significantly greater in the miR-4763-3p antagomir-treated group compared with the NC group (Figure 2B and E). These results suggest that miR-4763-3p antagomir treatment may rescue neuronal loss and damage in the hippocampal CA1 and CA3 regions of AD-MCI mice and ameliorate cognitive decline. In addition, since the normal structure of hippocampal synapses is fundamental to learning and memory, Golgi staining was used to evaluate the morphology of brain dendrites and synapses. The results showed that miR-4763-3p antagomir treatment significantly increased the length and density of the dendritic spines, while the dendritic spines in the miR-4763-3p agomir group were relatively disorganized (Figure 2F, Figure S2A). Moreover, Sholl analysis revealed that the dendrites of the miR-4763-3p agomir group exhibited the lowest complexity, and miR-4763-3p antagomir treatment significantly increased the complexity of dendrites (Figure 2F, G) and the percentage of mushroom-type spines (Figure 2H, I). Finally, the analysis of synaptic proteins revealed that the miR-4763-3p agomir reduced GLUR1/2 expression, while the antagomir increased GLUR1/2 expression (Figure S2B, C). These data suggest that miR-4763-3p antagomir treatment significantly rescues neuronal loss and reshaped synaptic morphology in AD-MCI mice, which may be the underlying mechanism for the restoration of cognition and enhancement of learning and memory.

### Bioinformatics analysis of the differential expression of miRNAs and related biochemical pathways in AD-MCI

To further explore the underlying molecular mechanisms and downstream signaling pathways involved in the regulation of miR-4763-3p, RNA-seq analysis of the hippocampal region of AD-MCI mice was performed following stereotactic injection of the miR-4763-3p antagomir or NC (Figure S2D). The results revealed 430 upregulated genes and 48 downregulated genes in comparison with those in the NC group (Figure S2E). Functional enrichment analysis of the DEGs revealed enrichment mainly in the immune system, phagosomes, the PI3K-AKT signaling pathway, apoptosis, and the mTOR signaling pathway (Figure S2F, G and Figure 2J). MiR-4763-3p antagomir mainly upregulated genes associated with membrane microdomains and membrane rafts (Figure 2K). To determine the relationship between the regulatory role of miR-4763-3p and the pathological mechanisms of AD, we further analyzed the DEGs between publicly available datasets for AD patients and healthy controls. Analysis of the raw RNA-seq data (GSE173955) using FDR/adj P < 0.05 and |log2FC|>1.5 identified 1429 DEGs between the AD group and the control group, of which 585 were upregulated and 844 were downregulated in the AD group (Figure S2G). The DEGs were mainly enriched in synapse organization, regulation of transporter activity, cognition (Figure S2G) and the mTOR signaling pathway (Figure 2L). Therefore, miR-4763-3p antagomir may influence the PI3K or mTOR signaling pathway, potentially improving the immune microenvironment in the brain, managing phagocytosis, and ameliorating the loss of synaptic function and neuron count in the brain.

### *ATP11A* is a target gene of miR-4763-3p in neurons

**Figure 3:**
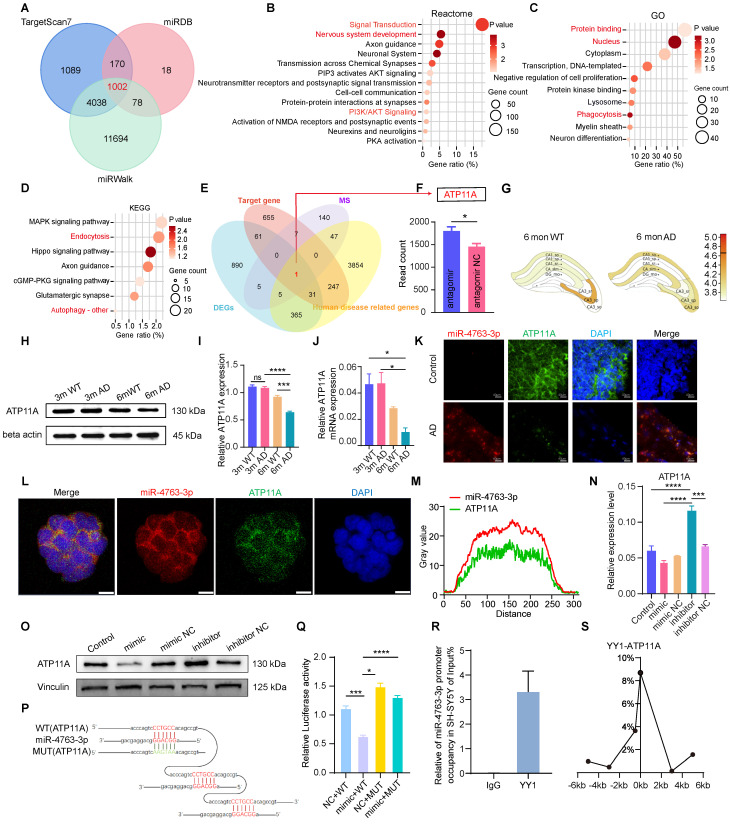
**MiR-4763-3p targets ATP11A, and the transcription initiation site is regulated by the transcription factor YY1.** A: Venn diagrams were generated using the target genes to predict miR-4763-3p. The data were obtained from the TargetScan7, miRDB, and miRWalk databases. B: Reactome enrichment analysis of the target genes. C: GO enrichment analysis of the target genes. D: KEGG enrichment analysis of the target genes. E: Venn diagrams were generated using RNA-seq-determined differentially expressed genes, miR-4763-3p predicted target genes, mass spectrometry results (SY5Y differentially transfected with miR-4763-3p inhibitor and NC), and human disease-related genes. F: The histogram shows the level of ATP11A expression in the hippocampus. G: Differential expression of ATP11A in different brain regions according to the AlzMap database. H-J: Protein and mRNA expression of ATP11A in the hippocampus of WT and 3×Tg mice at 3 and 6 months of age and a diagram of the statistical analysis. K: FISH and IF images of ATP11A (green) and miR-4763-3p (red) in the hippocampus of AD patients and normal controls were obtained. Nuclei are stained with DAPI (blue). Scale bar, 40 μm. L: FISH and IF staining of ATP11A (green) and miR-4763-3p (red) in SH-SY5Y cells. Nuclei are stained with DAPI (blue). Scale bar, 10 μm. M: Colocalization of miR-4763-3p and ATP11A in SH-SY5Y cells. N, O: Immunoblotting was used to evaluate the expression level of ATP11A in SH-SY5Y cells transfected with the miR-4763-3p mimic or inhibitor and NC. P: The predicted binding site between miR-4763-3p and wild-type ATP11A (ATP11A-WT) or between miR-4763-3p and mutant ATP11A (ATP11A-MUT). Q: Dual-luciferase activity in HEK-293T cells cotransfected with ATP11A-WT or ATP11A-MUT and the miR-4763-3p mimic or miR-4763-3p mimic NC. R: ChIP assays were carried out in SH-SY5Y cells using an antibody against YY1, and IgG was used as a negative control. The enrichment of YY1 binding to the miR-4763-3p promoter was quantified using qPCR. S: ChIP‒qPCR analysis of the association between YY1 and the promoter region of ATP11A in SH-SY5Y cells. The data are expressed as the mean ± SEM of three independent experiments. *P < 0.05; **P < 0.01; ***P < 0.001; ****P < 0.0001.

To verify our aforementioned hypothesis, bioinformatics analysis was conducted using online databases to predict the target genes of miR-4763-3p, with a total of 1002 (Figure 3A). Functional enrichment analysis indicated that the predicted target genes were involved mainly in nervous system development, PI3K/AKT signaling (Figure 3B), phagocytosis (Figure 3C) and autophagy (Figure 3D). Consistent findings from bioinformatics analysis and mice hippocampal sequencing analysis highlighted the significant involvement of autophagy and phagocytosis in the learning and memory impairments observed in neurodegenerative diseases such as AD. Further investigation revealed only ATP11A at the intersection of five datasets: mice hippocampus sequencing data DEGs, predicted miR-4763-3p target genes, mass spectrometry results (SY5Y differentially transfected with miR-4763-3p inhibitor and NC), and human disease-related genes (Figure 3E). ATP11A is closely related to the nervous system and immunity 26-28 and was significantly upregulated in the hippocampal region of mice treated with the miR-4763-3p antagomir (Figure 3F). *Atp11a* expression level in the CA1 brain region of the hippocampus decreased in AD-MCI mice at 6 months of age (Figure 3G, Figure S3A, B). Therefore, we hypothesized that Atp11a may be closely related to the decline in learning and memory abilities and cognitive impairment observed in early AD. We extracted RNA and protein from the hippocampus of 3-month-old and 6-month-old WT or AD-MCI mice for detection and found that the expression of ATP11A in 6-month-old AD-MCI mice was lower than that in WT mice (Figure 3H-J). Compared with the control group significant downregulation of ATP11A and elevated miR-4763-3p expression were observed in the hippocampus of patients diagnosed with AD and were found to be colocalized with ATP11A both in the brain tissue and cell line (Figure 3K-M), suggesting that there may be a close relationship between the two (Figure 3L, M). Subsequently, RT‒qPCR and western blotting of cells and tissues results demonstrated that miR-4763-3p inhibited the expression of ATP11A at both the transcriptional and translational levels and that ATP11A expression was restored following treatment with the miR-4763-3p inhibitor. In addition, this mode of action also affects ATP11A expression *in vivo* (Figure 3N, O; Figure S3C-E).

To verify the targeted binding between miR-4763-3p and ATP11A, RNAhybrid software was used to predict the binding sequence between miRNA and ATP11A. According to the predicted targeted binding sequence and the binding free energy analysis (Figure S3F, G), a mutant of ATP11A with a disrupted miR-4763-3p binding sequence was constructed. The sequencing results of the ATP11A mutant are shown in Figure S3H and I, and a schematic depicting the targeted binding mode between miR-4763-3p and WT or mutant ATP11A is illustrated in Figure 3P. HEK293T cells were cotransfected with ATP11A-WT or ATP11A-MUT and the miRNA mimic or NC. Compared with cells transfected with ATP11A-MUT, the miRNA mimic significantly reduced the luciferase activity in cells transfected with ATP11A-WT, indicating that miR-4763-3p inhibited the activity of the reporter gene and thus targeted regulated ATP11A (Figure 3Q). After revealing the targeting relationship between ATP11A and miR-4763-3p, we further determined the cognition-related functions of ATP11A. ShATP11A mice had showed shorter dwell times and shorter distances to the novel arm in the Y maze (Figure S4A-C), and the dwell times and distances explored around novel objects were significantly lower than those of NC mice (Figure S4D-F). In the Morris water maze test, the swimming time in the target quadrant was significantly lower than that in the NC group (Figure S4G, H), demonstrating that ATP11A deficiency led to learning and memory deficits in mice and that ATP11A, a target gene of miR-4763-3p, may play an important role in the process of AD disease.

In general, miRNAs act in mammals by not fully binding to target genes, thereby inhibiting their translation 29-31. However, our research findings demonstrated that miR-4763-3p also influences the transcription of ATP11A, which provoked our curiosity. Consequently, we conducted further investigations to explore the regulatory mechanisms underlying the transcription of miR-4763-3p. Considering that transcription factors (TFs) often play crucial roles in the mode of action of miRNAs 32-34, we employed gene function screening to predict the upstream TFs of miR-4763-3p and identified YY1 as a potential candidate (Figure S4I). Research has indicated that YY1 plays a vital role in phagocytosis and suppresses cytokine production and inflammation 35, providing a therapeutic target for the prevention and treatment of AD. Our experimental results revealed significant downregulation of YY1 expression in the hippocampus of AD-MCI patients compared to control subjects (Figure S4J). Based on the potential regulatory mechanism of YY1, the ChIP assay confirmed that YY1 could bind to the miR-4763-3p promoter in SH-SY5Y cells (Figure 3R), suggesting that YY1 can transcriptionally regulate miR-4763-3p expression. Inhibition of YY1 via siRNA upregulated the miR-4763-3p level (Figure S4K, L), but miR-4763-3p expression had no significant effect on YY1, demonstrating that YY1 may act only as an upstream regulatory element of miR-4763-3p. ChIP-qPCR was used to confirm that YY1 binds to the ATP11A promoter, initiating transcription in SH-SY5Y cells (Figure 3S). ATP11A expression was significantly downregulated after transfection with siYY1 (Figure S4M). In summary, our data revealed a feedforward regulatory mechanism of YY1-miR-4763-3p-ATP11A, which may play an important role in the nervous system of AD patients.

### MiR-4763-3p antagomir targets ATP11A to reverse early apoptosis in neurons by regulating PS flipping

**Figure 4:**
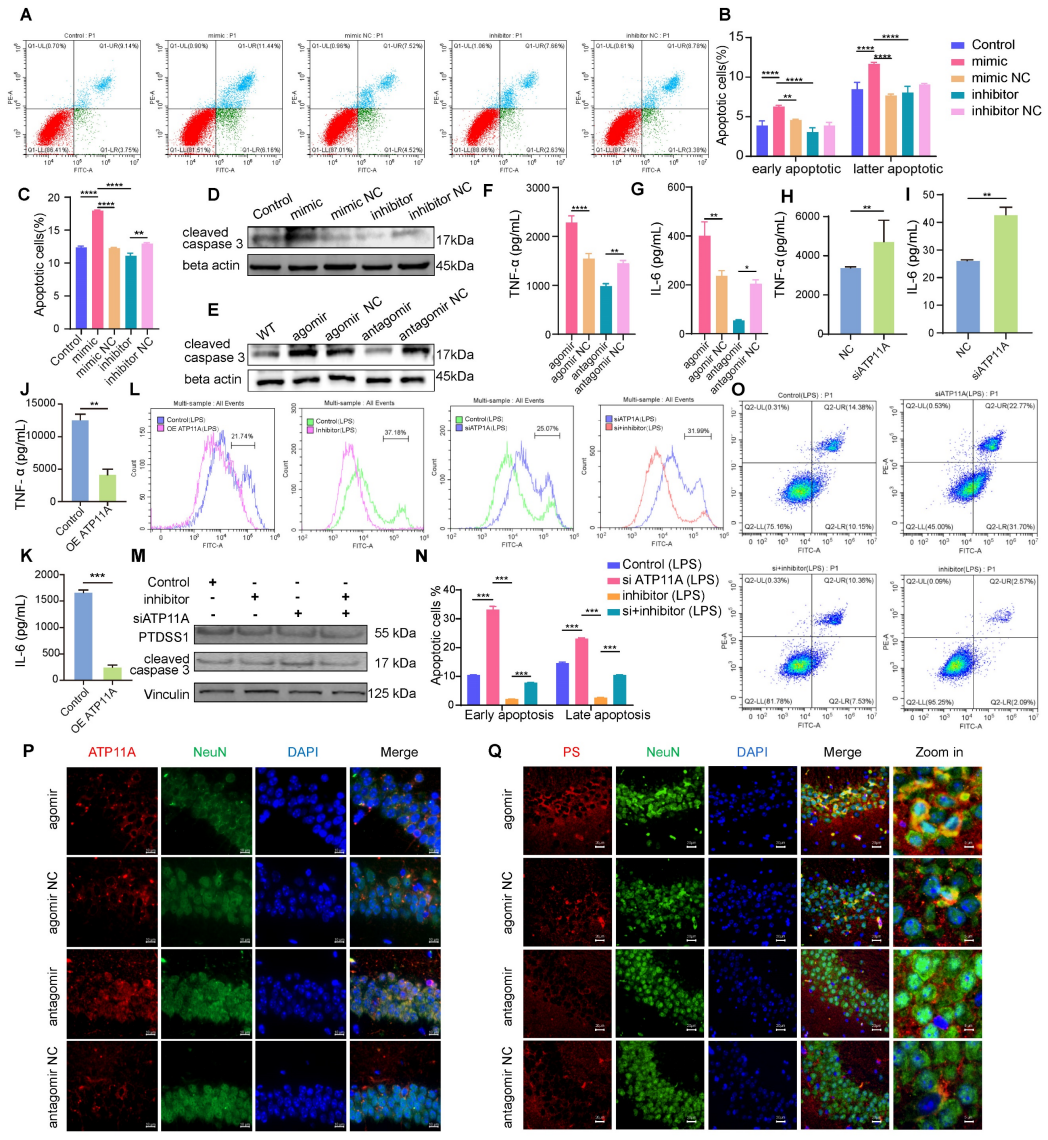
** MiR-4763-3p antagomir targets ATP11A to rescue early apoptosis in neurons by regulating PS flipping.** A: Flow cytometry analysis of the apoptotic rate of SH-SY5Y cells transfected with the miR-4763-3p mimic, mimic NC, inhibitor, and inhibitor NC and control cells. B, C: The histograms show the proportions of early, late and all apoptotic cells. D, E: Immunoblotting of cleaved caspase 3. F, G: The histograms of IL-6 and TNF-α in the hippocampus of mice injected with the miR-4763-3p agomir, agomir NC, antagomir and antagomir NC were analyzed by ELISA (n=3). H-K: ELISA measurement of plasma IL-6 and TNF-α levels in SH-SY5Y cells transfected with NC or overexpressing ATP11A (OE ATP11A) or siATP11A. L: Flow cytometry analysis of PS exposure in SH-SY5Y cells transfected with the miR-4763-3p inhibitor, siATP11A, or OE ATP11A and subsequently stimulated with LPS. O, N: Flow cytometry analysis of the apoptotic rate of SH-SY5Y cells transfected with the miR-4763-3p inhibitor or siATP11A and subsequently stimulated with LPS. The histogram shows the percentage of early and late apoptotic cells. M: Immunoblotting of PTDSS1 and cleaved caspase 3. Vinculin served as an internal reference protein. P: IF staining of NeuN (green) and ATP11A (red) in mouse brains from the antagomir NC, antagomir, agomir NC, and agomir groups. Nuclei were stained with DAPI (blue). Scale bar, 10 μm. Q: IF staining of NeuN (green) and PS (red) in mouse brains from the antagomir NC and antagomir groups. Nuclei were stained with DAPI (blue). Scale bar, 20 μm. The data are expressed as the mean ± SEM of three independent experiments. *P < 0.05; **P < 0.01; ***P < 0.001; ****P < 0.0001.

Based on our results in mouse hippocampal slices, we hypothesized that the miR-4763-3p antagomir may rescue neuronal loss; however, the specific mechanism is unclear. Currently known forms of regulated cell death include apoptosis, necrosis and autophagy 36. Based on RNA-seq data, it is reasonable to suggest that miR-4763-3p is closely related to cellular apoptosis. Flow cytometry was used to detect apoptosis in SH-SY5Y cells transfected with the miR-4763-3p mimic, inhibitor, corresponding NC or control. Annexin V+ PI-was defined as early apoptosis and Annexin V+ PI+ was defined as late apoptosis 37. The results showed that compared with the NC, the miR-4763-3p mimic increased the levels of early and late apoptosis, while the miR-4763-3p inhibitor significantly reduced the proportion of early apoptotic cells (Figure 4A-C). Immunofluorescence and western blotting data revealed that treatment with the miR-4763-3p antagomir/inhibitor resulted in a lower level of cleaved caspase 3, and the cleaved caspase 3 expression level in the agomir or mimic treatment group was increased (Figure 4D, E; Figure S5A, B). We hypothesize that the mechanism by which miR-4763-3p affects apoptosis is related to brain inflammation; therefore, ELISA, IHC and qPCR were performed to evaluate the expression of the inflammatory factors IL-6 and TNF-α in the mouse brain. The results revealed that the miR-4763-3p agomir significantly increased the expression levels of IL-6 and TNF-α, while treatment with the miR-4763-3p antagomir reduced brain inflammation (Figure 4F, G, Figure S5C-G). We also further explored the sequencing data of miR-antagomir and AD-MCI-NC and found that IL-34 is significantly affected by miR-4763-3p, a cytokine that is closely associated with the clearance of Aβ and stimulates the release of proinflammatory factors from macrophages. The qPCR results in brain tissue showed that compared with the control, the miR-4763-3p antagomir significantly increased the expression of IL-34 in brain tissue. Therefore, we speculate that the miR-4763-3p antagomir improves the inflammatory environment in the brain by reducing the expression of IL-34 and reducing the release of inflammatory factors from macrophages (Figure S5 H, I).

We hypothesized that the target gene of miR-4763-3p, ATP11A, might be related to inflammation in the brain. ELISA data demonstrated that siATP11A significantly increased the expression of IL-6 and TNF-α, while ATP11A overexpression significantly reduced the expression levels of IL-6 and TNF-α, indicating that ATP11A overexpression may alleviate brain inflammation (Figure 4H-K). ATP11A is generally present in plasma membranes, and studies have shown that it has phospholipid flipping activity 38; therefore, this study further explored whether ATP11A may play a role in phospholipid flipping in neuronal cell membranes. ATP11A was significantly upregulated in the CA1 and CA3 regions of the hippocampus in the miR-4763-3p antagomir-treated group, while its expression was relatively low in the miR-4763-3p agomir-treated group (Figure 4P). Notably, ATP11A was colocalized with neurons in the hippocampus, and its abundance and density were greater in the brains of mice treated with the miR-4763-3p antagomir. This may be more beneficial for its function: flipping the membrane of phosphatidylserine (PS). Flow cytometry analysis was performed in SH-SY5Y cells stimulated with LPS using Annexin V-FITC staining to measure phosphatidylserine expression levels, and the shift in the X-axis represents the effect of ATP11A on extracellular PS. The results demonstrated that overexpression of ATP11A and treatment with the miR-4763-3p inhibitor significantly decreased the level of extracellular PS (Figure 4L), while siATP11A treatment significantly increased PS in the outer leaflet of the plasma membrane of neurons. Moreover, compared with siATP11A treatment alone, cotransfection of cells with the miR-4763-3p inhibitor resulted in a significant decrease in the outer member PS level, especially following LPS treatment (Figure 4L; Figure S6A-C). These results demonstrate that miR-4763-3p plays an important role in stabilizing the phospholipid balance inside and outside the cell membrane under inflammatory conditions. Immunofluorescence staining of brain tissue revealed much stronger PS staining in the NC group than in the miR-4763-3p antagomir-treated group (Figure 4Q), indicating that the miR-4763-3p antagomir stimulates ATP11A to inwardly flip PS into the cytoplasm, reducing the early apoptotic "eat me" signal on the cell surface. Flow cytometry analysis confirmed that siATP11A significantly increased apoptosis, particularly in the early stages. Conversely, treatment with an inhibitor of miR-4763-3p effectively mitigated both the early and late stages of neuronal apoptosis induced by ATP11A deficiency (Figure 4O, N; Figure S6D-H). Additionally, this regulatory mechanism of apoptosis did not affect PS synthase expression levels, which demonstrated that the miR-4763-3p inhibitor affected only PS flipping activity rather than synthesis (Figure 4M; Figure S6I, J). All in all the miR-4763-3p inhibitor can stimulate PS flipping into cells by increasing ATP11A levels. This flip can greatly reduce the recognition of "eat me" signals on the surface of glial cells under inflammatory conditions, reshaping the inflammatory environment in the brain; consequently, this mechanism reduces the phagocytic impact of glial cells on neuronal synapses and cell bodies, thus achieving the desired outcome of reducing early apoptosis of neurons.

### MiR-4763-3p antagomir/ATP11A increases autophagy levels in neurons

**Figure 5:**
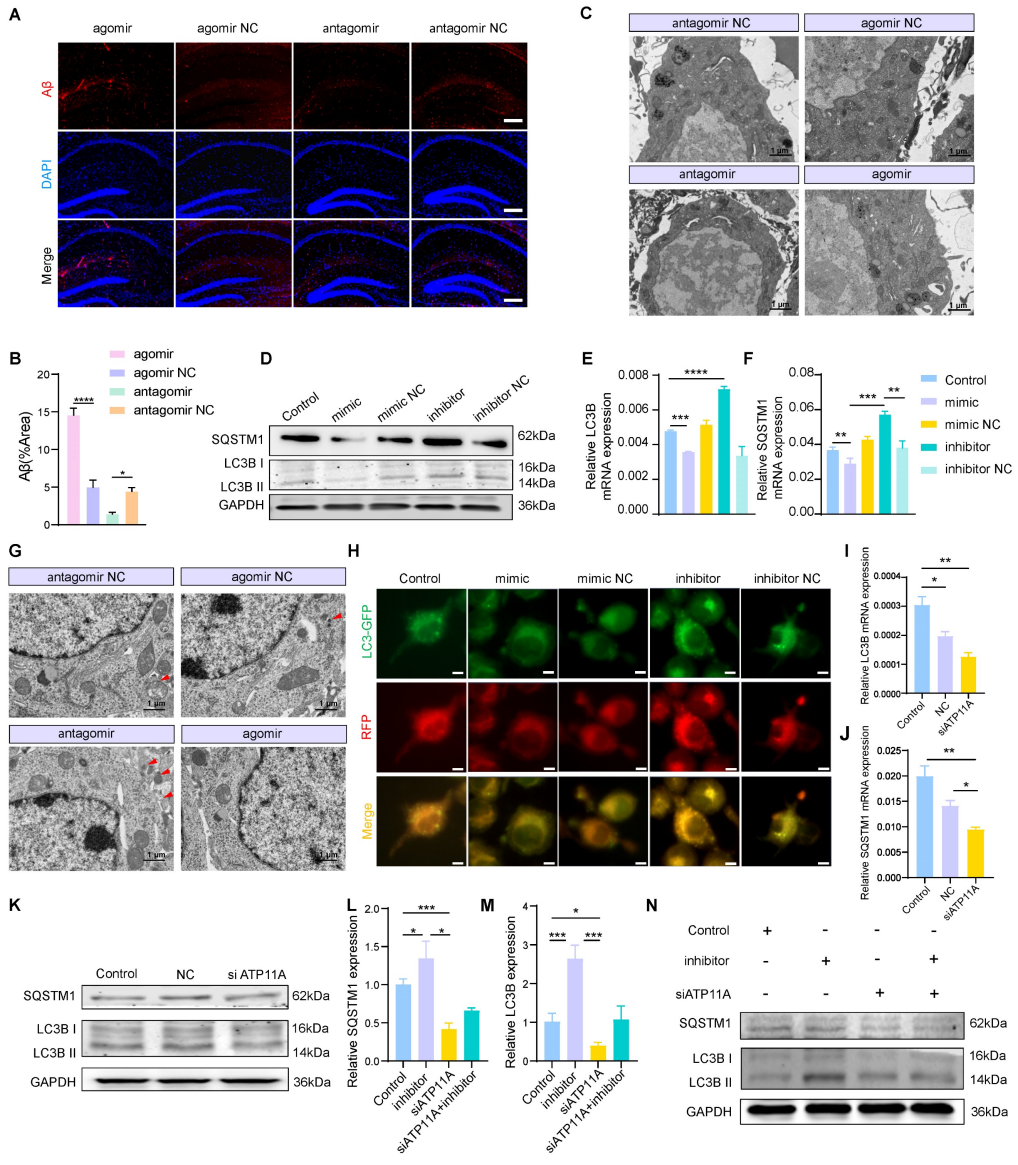
**MiR-4763-3p antagomir/ATP11A increases autophagic flux in neurons.** A, B: IF staining of Aβ in the mouse hippocampus. The graph displays the results of quantitative Aβ staining (n=3). Scale bar, 20 μm. C: Transmission electron microscopy (TEM) of the ultrastructure of the mouse hippocampus (the red arrow indicates lipofuscin). Scale bar, 1 μm, 5 μm. D-F: RT‒qPCR and immunoblotting of autophagic flux markers in SH-SY5Y cells transfected with the miR-4763-3p mimic, mimic NC, inhibitor, inhibitor NC or control. G: TEM of neuronal autophagosomes in the mouse hippocampus (red arrows indicate autophagosomes). Scale bar, 1 μm, 5 μm. H: IF staining of LC3B-GFP (green) and RFP (red) in SH-SY5Y cells from the control, mimic NC, mimic, inhibitor NC, and inhibitor groups. Scale bar, 10 μm. I-K: RT‒qPCR and immunoblotting of autophagic flux markers in SH-SY5Y cells transfected with control, NC or siATP11A. L‒N: RT‒qPCR and immunoblotting of autophagic flux markers in SH-SY5Y cells transfected with control, inhibitor and inhibitor cotransfected with siATP11A. The data are expressed as the mean ± SEM of three independent experiments. *P < 0.05; **P < 0.01; ***P < 0.001; ****P < 0.0001.

Since the miR-4763-3p antagomir improves PS flipping under inflammatory conditions and reverses the occurrence of apoptosis, whether cellular homeostasis within neurons is also improved is unclear. Revisiting RNA-seq data and functional enrichment data for miR-4763-3p target genes points to autophagy (Figure 3D). To investigate the involvement of miR-4763-3p in the regulation of autophagy and its potential regulatory role in early Aβ deposition, we conducted IF staining of hippocampal tissue sections from the stereotactic injections of miR-4763-3p antagomir, agomir, antagomir NC, and agomir NC in AD-MCI mice. The results showed that the NC group, particularly the miR-4763-3p agomir-treated group, exhibited significant Aβ deposition, whereas the miR-4763-3p antagomir-treated group exhibited almost no Aβ deposition (Figure 5A, B). We speculated that the miR-4763-3p antagomir may reduce Aβ deposition during the early stages of the disease by facilitating autophagy or phagocytosis. TEM was used to evaluate changes in intracellular structures in the hippocampal region of AD-MCI mice following treatment with NC or the miR-4763-3p antagomir, revealing greater amounts of lipofuscin in the neurons of mice in the NC and agomir-treated groups, antagomir group lower (Figure 5C). Excess lipofuscin indicates incomplete elimination of lipids and/or misfolded proteins in the cell body, suggesting insufficient lysosomal activity and excessive accumulation of metabolic waste, which may further impair neuronal function or cause neuronal death 39, 40. However, the amount and size of lipofuscin were significantly reduced in the miR-4763-3p antagomir-treated group, which led us to believe that the miR-4763-3p antagomir may improve the intracellular environment of neurons by facilitating autophagy or lysosomal phagocytosis. During the early stages of autophagy, SQSTM1 functions as a selective autophagy receptor and serves as an important protein marker. Studies have demonstrated that SQSTM1 binds to arginine-modified substrates and induces autophagy. The depletion of SQSTM1 inhibited the recruitment of LC3 to autophagosomes, hindered the formation of autophagosomes within cells, and may impair autophagy 41-43.

The basal levels of autophagy and lysosomal biogenesis can be reflected by the expression levels of LC3B II/I and SQSTM1. Compared with those in the control group, the miR-4763-3p inhibitor significantly increased the mRNA expression levels of LC3B and SQSTM1, as well as the protein expression levels of LC3B II/I and SQSTM1, whereas the mimic decreased the expression of these genes. The same trend was observed in the hippocampal CA1 and CA3 regions of mice following injection (Figure 5D-F; Figure S7A-C). Transmission electron microscopy revealed a significant increase in the number of autophagosomes in hippocampal neurons after miR-4763-3p antagomir treatment compared with those in the NC- and miR-4763-3p agomir-treated groups (Figure 5G). In addition, SH-SY5Y cells were transfected with LC3-GFP-RFP to determine the effect of miR-4763-3p on autophagic flux. GFP is an acid-sensitive protein that emits yellow fluorescence in autophagosomes and red fluorescence in autolysosomes. After miR-4763-3p inhibitor treatment, the number of red and yellow fluorescent intracellular structures increased significantly in comparison with that in the control group, indicating an increase in autophagic flux. In contrast, only green fluorescent structures were observed following miR-4763-3p mimic treatment, suggesting that miR-4763-3p may block the fusion of autophagosomes with lysosomes (Figure 5H). To further elucidate the underlying mechanism by which miR-4763-3p regulates autophagic flux, we hypothesized that its target gene ATP11A may play a role in modulating the expression of SQSTM1; therefore, we investigated the impact of ATP11A on SQSTM1. The results demonstrated that the levels of LC3B and SQSTM1 decreased after treatment with siATP11A compared with those in the control and NC groups (Figure 5I-K; Figure S7D, E). Interestingly, cotransfection of miR-4763-3p inhibitor and siATP11A restored autophagic flux (Figure 5L-N; Figure S7F, G). These data suggested that miR-4763-3p can target ATP11A to inhibit the fusion of autophagosomes with lysosomes, thereby reducing autophagy levels. The miR-4763-3p antagomir can increase autophagic flux in the brain to some extent and clear the excessive deposition of Aβ and lipofuscin in a timely manner, improving the brain environment and restoring spatial memory and cognitive abilities in AD-MCI mice.

### Inhibition of the PI3K/AKT/mTOR/Bcl2 axis results in crosstalk between autophagy and apoptosis

**Figure 6:**
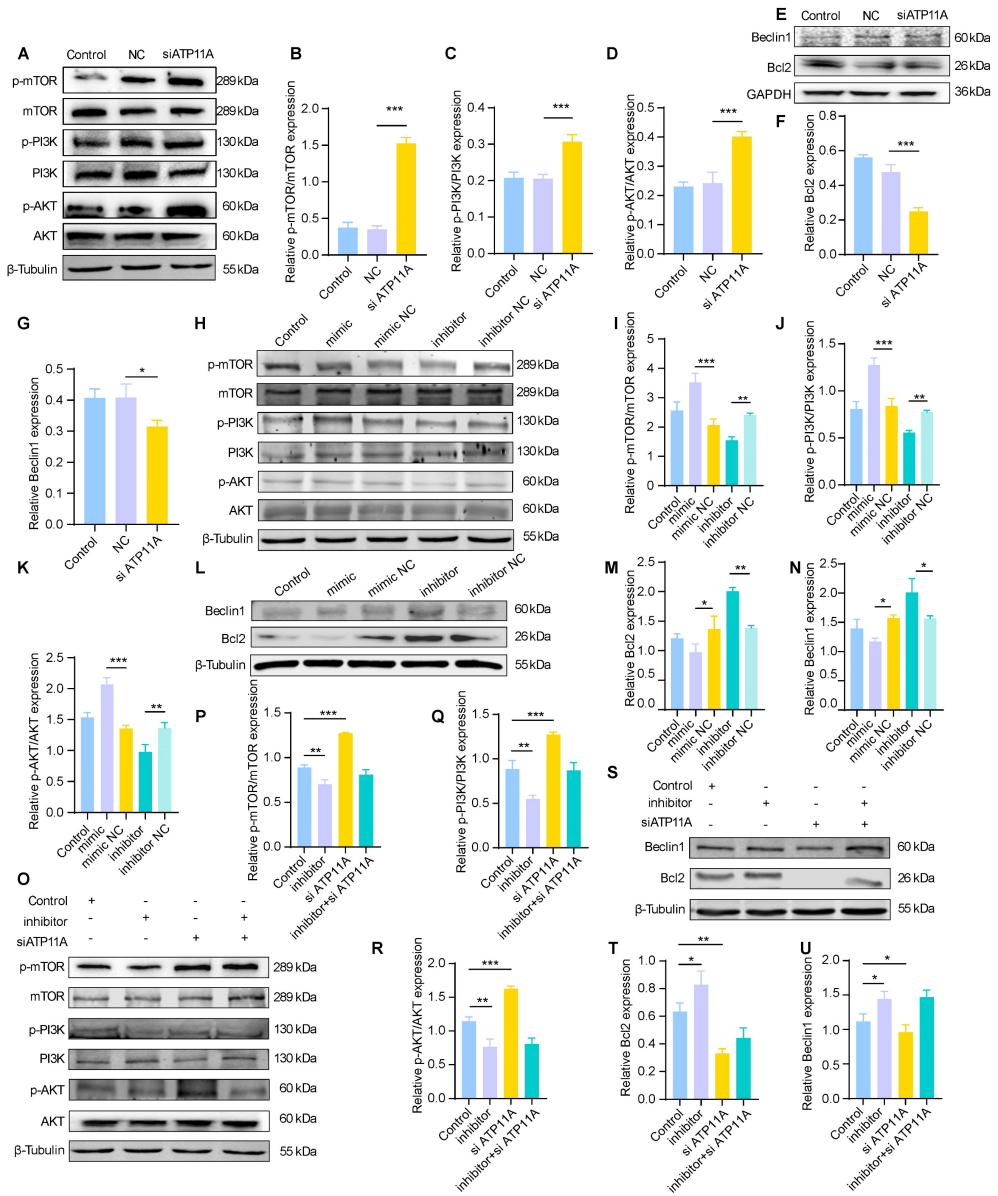
** The miR-4763-3p antagomir/ATP11A inhibits the PI3K/AKT/mTOR signaling pathway to mediate crosstalk between the autophagic protein Beclin1 and the apoptotic protein Bcl2.** A: Immunoblotting of p-mTOR, mTOR, p-PI3K, PI3K, p-AKT and AKT in SH-SY5Y cells transfected with control, NC and siATP11A. β-Tubulin served as an internal reference protein. B-D: Quantitative analysis of p-mTOR/mTOR, p-PI3K/PI3K and p-AKT/AKT protein expression. E-G: Immunoblotting of Beclin1 and Bcl2 in SH-SY5Y cells transfected with control, NC or siATP11A. GAPDH served as an internal reference protein. Quantitative analysis was performed on the obtained data. H-K: Immunoblotting of p-mTOR, mTOR, p-PI3K, PI3K, p-AKT and AKT in SH-SY5Y cells transfected with control, mimic, mimic NC, inhibitor or inhibitor NC. β-Tubulin served as an internal reference protein (H). Quantitative analysis was performed on the obtained data (I-K). L-N: Immunoblotting of Beclin1 and Bcl2 in SH-SY5Y cells transfected with control, mimic, mimic NC, inhibitor or inhibitor NC. β-Tubulin served as an internal reference protein. Quantitative analysis was performed on the obtained data. O-U: Immunoblotting of SH-SY5Y cells cotransfected with siATP11A and control, inhibitor or inhibitor. β-Tubulin served as an internal reference protein. Quantitative analysis was performed on the obtained data. The data are expressed as the mean ± SEM of three independent experiments. *P < 0.05; **P < 0.01; ***P < 0.001; ****P < 0.0001.

Autophagy and apoptosis exhibit a dynamic balance in the brain, which is mediated by signaling pathways. To further investigate the regulatory mechanism of the miR-4763-3p antagomir/ATP11A, we hypothesized that miR-4763-3p is likely involved in modulating the PI3K/AKT/mTOR signaling pathway. This hypothesis is based on combined KEGG analysis of sequencing data from the hippocampal region of AD-MCI mice treated with miR-4763-3p antagomir, reactome analysis of target genes regulated by miR-4763-3p, and KEGG analysis comparing DEGs between AD patients and normal controls. To verify this hypothesis, we examined the protein expression of p-mTOR, mTOR, p-PI3K, PI3K, p-AKT, and AKT (Figure 6).

The immunoblotting results showed that p-mTOR/mTOR, p-PI3K/PI3K, and p-AKT/AKT were significantly upregulated in the siATP11A- and miR-4763-3p mimic-treated cells (Figure 6A-D, H-K; Figure S8A-C, F-H). In addition, Beclin1 and Bcl2 were downregulated following siATP11A and mimic treatment (Figure 6E-G, L-N; Figure S8D, E, I, J), indicating that activation of the PI3K/AKT/mTOR signaling pathway inhibited autophagy and activated the apoptotic pathway. The levels of p-mTOR/mTOR, p-PI3K/PI3K, and p-AKT/AKT were significantly decreased in SH-SY5Y cells treated with the miR-4763-3p inhibitor, indicating that the PI3K/AKT/mTOR signaling pathway was inhibited (Figure 6H-K, Figure S8F-H). Bcl2 and Beclin1 were also significantly increased in these cells, indicating that the antiapoptotic pathway was activated (Figure 6L-N, Figure S8I, J). Ultimately, cotransfection of siATP11A and the miR-4763-3p inhibitor had an appreciable ameliorating effect (Figure 6O-U; Figure S8K-O). Specifically, the PI3K/AKT/mTOR signaling pathway was activated following the administration of siATP11A compared with that in the control group. In addition, Bcl2 and Beclin1 expression was downregulated, which could be reversed by the introduction of the miR-4763-3p inhibitor. In conclusion, these findings confirmed that miR-4763-3p can target ATP11A to modulate the PI3K/AKT/mTOR/Bcl2 signaling pathway, thereby stabilizing the levels of autophagy and apoptosis in the brain and establishing specific cross-talk relationships.

## Discussion

Here, bioinformatics analysis revealed significant upregulation of miR-4763-3p in the serum of individuals with AD, and this upregulation was positively correlated with age. Subsequently, we determined that miR-4763-3p was highly expressed in the brains of AD patients and colocalized with the Aβ and tau proteins. Intracerebral stereotactic injection of a miR-4763-3p antagomir significantly improved learning and memory impairment and cognitive decline in an AD-MCI mouse model. We found that miR-4763-3p antagomir treatment improved the progressive loss of hippocampal neurons in AD-MCI mice, which was accompanied by an increase in the number of Nissl bodies, dendritic number and synaptic complexity. RNA-seq analysis of hippocampal tissue from control and miR-4763-3p antagomir-treated AD-MCI mice demonstrated potential involvement in regulating the immune system, phagosomes, and apoptosis. Subsequently, we identified the YY1-miR-4763-3p-ATP11A feedforward regulatory mechanism, which reduces the inflammatory environment in the brain and enhances the ability of ATP11A to inwardly flip PS in the neuronal cell membrane under inflammatory conditions. This reduces the level of early apoptosis and inflammation in neurons and regulates autophagy through the PI3K/AKT/mTOR/Bcl2 signaling pathway.

Late-stage AD is an irreversible progressive brain disorder characterized by memory loss and cognitive decline, which is accompanied by severe neuronal loss and widespread inflammation, and there is currently no effective cure 44-46. Therefore, early detection, prevention, and intervention strategies for the early stages of the disease are increasingly considered the keys to more effective management and treatment 47, 48. MCI is a transitional state between normal aging and dementia, during which cognitive impairment does not yet affect daily life; nevertheless, reports suggest that 15-20% of these patients subsequently develop AD 49-51. Some studies suggest that the failure of experimental disease treatments to date is due to their being conducted in patients who already meet the AD criteria, which may represent too late a time point 52, 53.

In this research, we leveraged clinical samples and animal experiments to identify the dysregulation of miR-4763-3p accompanying the progression of AD. Notably, in the context of the APOE high-risk factor, a positive correlation trend was observed between the age of onset of AD and the expression level of miR-4763-3p. Surprisingly, miR-4763-3p was highly expressed in the hippocampus of AD patients and appeared to colocalize with neurons, Aβ, and Tau, while its expression level in healthy controls was low. This finding suggested that miR-4763-3p may play an important role in the course of AD. Six-month-old 3×Tg mice exhibit early AD symptoms but no tau pathology, but Aβ deposition and synaptic dysfunction are already present, which manifests as early cognitive deficits, and these mice can be used as an AD-MCI model. Here, AD-MCI mice were subjected to stereotactic injection of a miR-4763-3p agomir or antagomir to explore their effects. Multiple behavioral experiments related to memory demonstrated that the miR-4763-3p antagomir effectively rescued the cognitive deficits of AD-MCI mice. Moreover, examination of hippocampal tissue revealed that the miR-4763-3p antagomir significantly restored the number and synaptic density of neurons and Nissl bodies. Therefore, the specific mechanism by which the miR-4763-3p antagomir rescued AD-MCI at an early stage was investigated.

The mode of action of miRNAs is generally considered to involve targeted inhibition of gene expression 54; thus, RNA-seq is undoubtedly the most direct method for revealing the mechanism of action of miR-4763-3p. The results showed that the target genes of miR-4763-3p are mainly involved in the immune system, phagocytosis, synapse organization, regulation of nervous system processes, cognition, and learning or memory in biological processes. We identified a direct target of miR-4763-3p, *ATP11A*, which encodes a P4-type ATPase that functions redundantly as a phospholipid flippase in the plasma membrane. Previous studies have shown that mutations in ATP11A cause developmental delays and neurological deterioration 55, and ATP11A participate in the formation of the syncytiotrophoblast layer during placental development 38. These observations suggest that ATP11A plays an important role in the development of the nervous system and may be closely related to the development or death of neurons.

The brains of AD patients are considered to have an inflammatory microenvironment, and the present study proposes an unknown regulatory mechanism between autophagy and apoptosis. The miR-4763-3p antagomir targets *ATP11A* to reduce the expression of inflammatory factors in the brain and enhances the ability of ATP11A to inwardly flip PS inside the neuronal cell membrane. This reduces the exposure of the "eat me" signal on the neuronal surface, thereby decreasing the recognition and phagocytosis of neurons by glial cells and reversing their early apoptosis. Moreover, homeostasis within neurons is improved, autophagy levels are increased to clear metabolic waste, brain lipofuscin levels and Aβ deposition are reduced, and a healthy microenvironment is restored within brain neurons.

Autophagy defects often occur in early AD-MCI, where abnormal protein aggregates and Aβ+ autophagic vacuoles (AVs) containing incompletely digested autophagic substrates accumulate in neurons. These effects are associated with an age-induced reduction in autophagy-related gene expression and late-onset AD 56-58. Therefore, autophagy dysfunction may act as an upstream event of AD amyloid pathology, rendering it an attractive target for therapeutic intervention 59 since maintaining the crosstalk balance between autophagic flux and apoptosis is particularly important for the treatment of nervous system diseases. The balance between autophagy and apoptosis is intricately regulated in brain tissues. Autophagy can reduce cellular apoptosis by eliminating damaged fragments or degraded subcellular components 60, 61. Studies have shown that activation of the TNF-α/TNFR1 signaling pathway in AD leads to the recruitment of RIPK1 by accumulated p62, which induces its oligomerization and results in necroptotic death of neurons 62. Autophagy also limits endoplasmic reticulum (ER) stress by degrading unfolded protein aggregates. Autophagy is regulated by various signaling pathways, among which mTOR, a protein kinase, senses the availability of cellular energy and regulates cell proliferation. Reports have shown excessive activation of mTOR signaling in specific brain regions of AD patients 63-65. Therefore, inhibition of the PI3K/AKT/mTOR pathway can activate autophagy, which is consistent with our functional enrichment and immunoblotting data. Inhibition of miR-4763-3p resulted in a significant increase in the expression levels of LC3B II/I and SQSTM1, as well as a notable reduction in the accumulation of Aβ and lipofuscin in the hippocampal region while the number of autophagosomes was increased. Taken together, these data indicated that the miR-4763-3p antagomir promoted autophagy by inhibiting the PI3K/AKT/mTOR/Bcl2 signaling pathway and reducing cell apoptosis, participates in the balance between brain autophagy and early neuronal apoptosis in AD-MCI mice.

Finally, there are some limitations in this paper. First, although a feedback loop mechanism involving YY1-miR-4763-3p-ATP11A was found in this study, the interaction between these two factors and the YY1 transcription of these two genes was not fully explored in this study, which may be a shortcoming of this study. In addition, ATP11A, the key target gene of this protein, is a phospholipid flipping enzyme. The effect of PS in this study was mainly to reduce the inflammatory microenvironment in the brain and flip PS into the inner membrane of nerve cells, thereby improving the level of inflammation in nerve cells and improving autophagy. Does PS play a role in autophagy in nerve cells? How PS, as a lipid structure, may affect autophagosome assembly may be worth further exploration. In addition, while we have contributed to understanding the role of miR-4763-3p in relevant therapeutic outcomes in mouse models, its preclinical application needs to be further explored. In future studies, it will be necessary to optimize the biologics of miR-4763-3p antagomirs to improve their stability and therapeutic efficacy *in vivo*.

In summary, we have identified a dysregulated miRNA in AD, whose suppression at an early stage can effectively mitigate cognitive decline. By forming a feedforward loop with YY1-ATP11A, miR-4763-3p modulates the mTOR signaling pathway within the inflammatory milieu of the brain, thereby maintaining a balance between apoptosis and autophagy, and efficiently remodels the microenvironment both inside and outside neurons. This finding points to a promising strategy for the treatment of AD-MCI patients.

## Supplementary Material

Supplementary figures and tables.
