# Supplementary material for: Inhibition of miR-4763-3p expression activates the PI3K/mTOR/Bcl2 autophagy signaling pathway to ameliorate cognitive decline
Source: Int J Biol Sci. 2024 Nov 4;20(15):5999–6017. doi: 10.7150/ijbs.103225 (PMC11628338; doi:10.7150/ijbs.103225)
Supplement: Supplementary file 1 — Supplementary figures and tables. [file ijbsv20p5999s1.pdf]

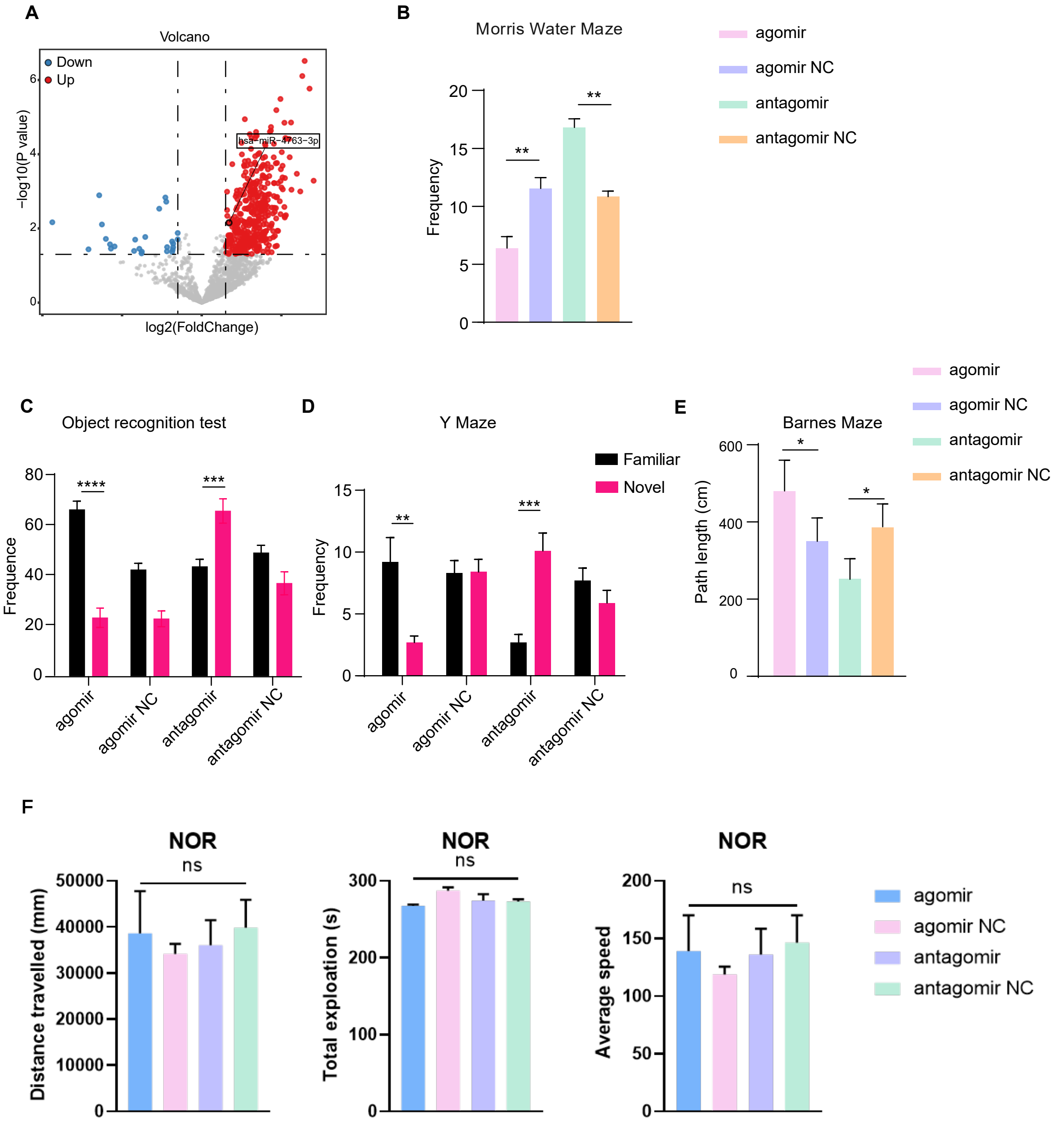

A
Volcano
Down
Up
hsa-miR-4763-3p
-log10(P value)
log2(FoldChange)
B
Morris Water Maze
Frequency
agomir
agomir NC
antagomir
antagomir NC
C
Object recognition test
Frequence
D
Y Maze
Familiar
Novel
Frequency
E
Barnes Maze
Path length (cm)
F
NOR
ns
Distance travelled (mm)
Total exploation (s)
Average speed

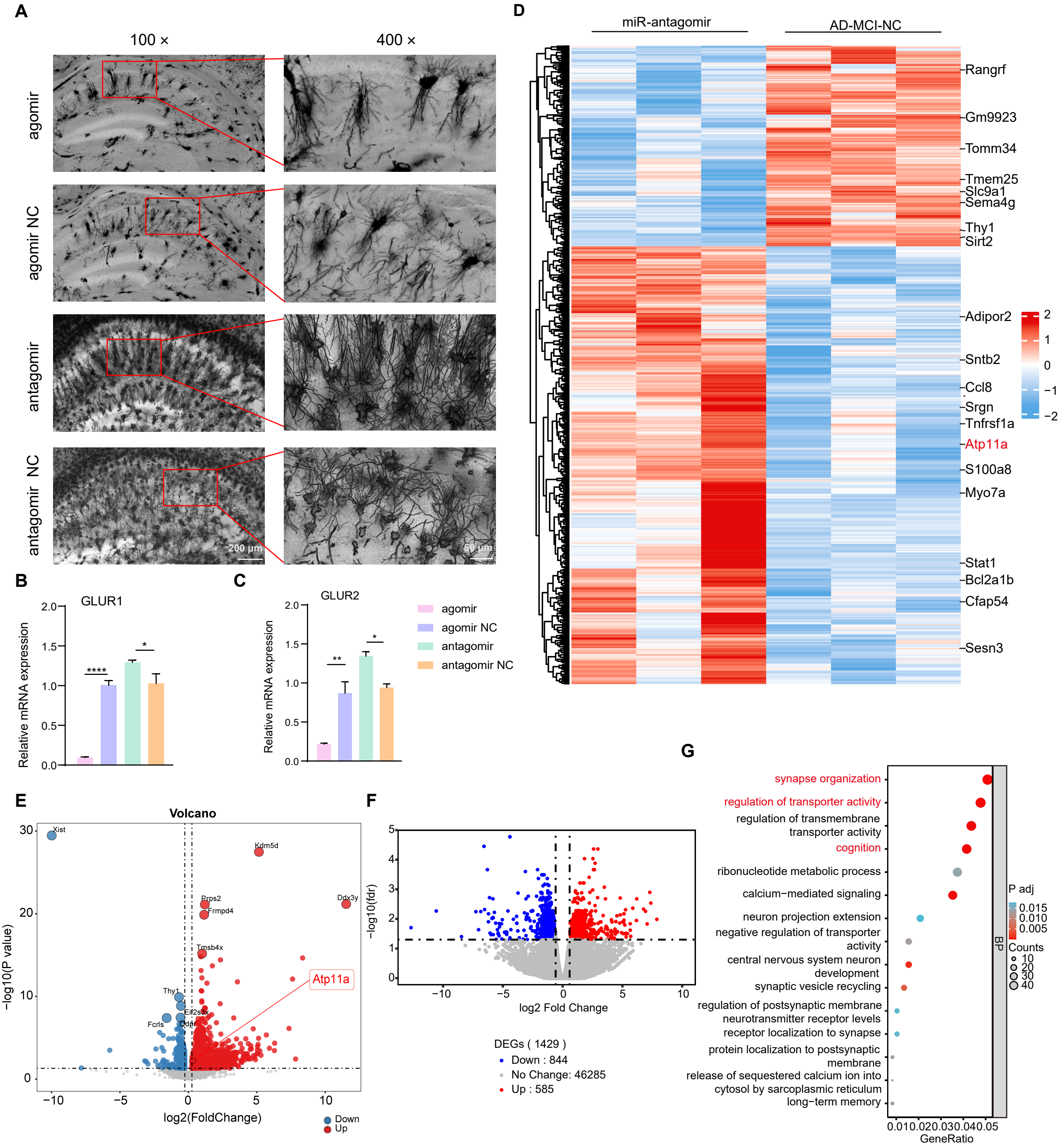
A
100 ×
400 ×
agomir
agomir NC
antagomir
antagomir NC
200 μm
50 μm
B
GLUR1
Relative mRNA expression
2.0
1.5
1.0
0.5
0.0
****
*
C
GLUR2
Relative mRNA expression
2.0
1.5
1.0
0.5
0.0
**
*
agomir
agomir NC
antagomir
antagomir NC
D
miR-antagomir
AD-MCI-NC
Rangrf
Gm9923
Tomm34
Tmem25
Slc9a1
Sema4g
Thy1
Sirt2
Adipor2
Sntb2
Ccl8
Srgn
Tnfrsf1a
Atp11a
S100a8
Myo7a
Stat1
Bcl2a1b
Cfap54
Sesn3
2
1
0
−1
−2
E
Volcano
−log10(P value)
log2(FoldChange)
Xist
Kdm5d
Prps2
Frmpd4
Ddx3y
Tmsb4x
Atp11a
Thy1
Eif2s3x
Fcrls
Down
Up
F
−log10(fdr)
log2 Fold Change
DEGs (1429)
Down : 844
No Change: 46285
Up : 585
G
synapse organization
regulation of transporter activity
regulation of transmembrane transporter activity
cognition
ribonucleotide metabolic process
calcium−mediated signaling
neuron projection extension
negative regulation of transporter activity
central nervous system neuron development
synaptic vesicle recycling
regulation of postsynaptic membrane neurotransmitter receptor levels
receptor localization to synapse
protein localization to postsynaptic membrane
release of sequestered calcium ion into cytosol by sarcoplasmic reticulum
long−term memory
BP
P adj
0.015
0.010
0.005
Counts
10
20
30
40
GeneRatio
0.010.020.030.040.05

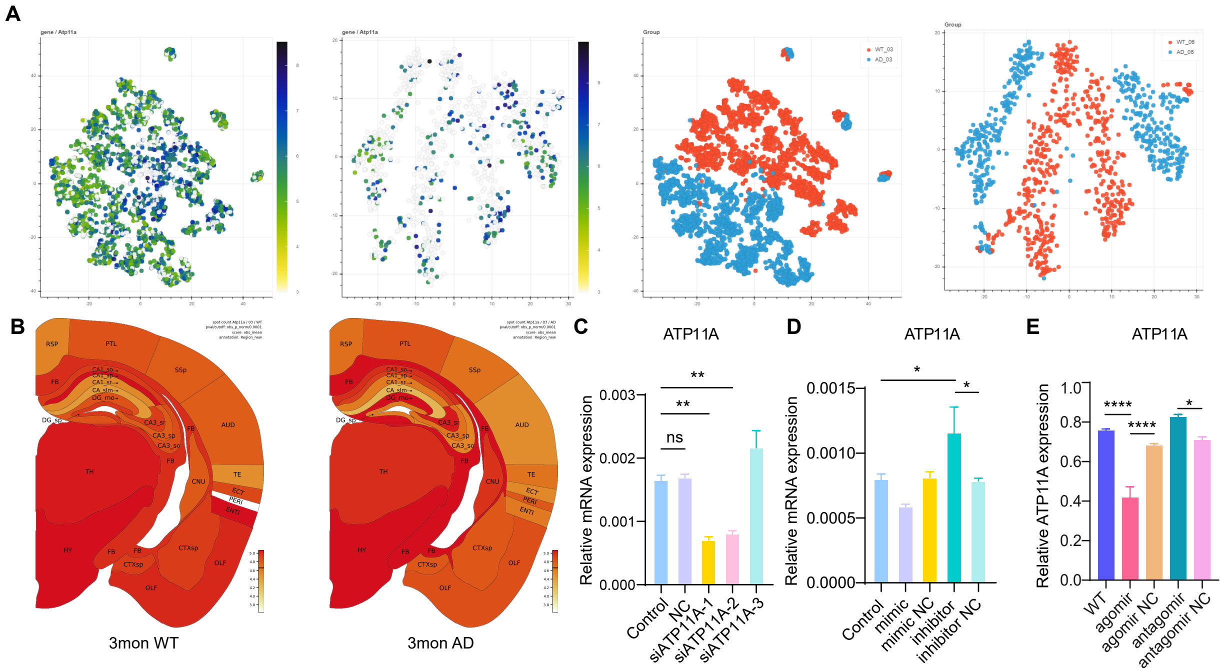

F

```
miRNA : hsa-miR-4763-3p
target 5' G      AA    C      CAA    A     G 3'
           CCGCCU  AGC CCAGU    CCC GCCU
           GGCGGG  UCG GGUCG    GGG CGGA
miRNA  3'              U            A      5'

miRNA : hsa-miR-4763-3p
target 5' G       U   UU G             C 3'
           CCGCCC GGC  G CAG CCCUGCC
           GGCGGG UCG  U GUC GGGACGG
miRNA  3'              G   G         A 5'

miRNA : hsa-miR-4763-3p
target 5' A         GCUU             G 3'
           CG CCGGCA    CCAGCCC UGCC
           GC GGUCGU    GGUCGGG ACGG
miRNA  3' G  G                 G     A 5'

miRNA : hsa-miR-4763-3p
target 5'  G     CU   UGAUUCACAC           A 3'
            GCCUG  GCA          CCAGUCCCUGCC
            CGGGU  CGU          GGUCGGGGACGG
miRNA  3' GG                               A 5'

miRNA : hsa-miR-4763-3p
target 5'  G     CU   UGAUUCACAC           A 3'
            GCCUG  GCA          CCAGUCCCUGCC
            CGGGU  CGU          GGUCGGGGACGG
miRNA  3' GG                               A 5'
```

G

miRNA	Common target	position	MFE (kcal/mol)	p-value
hsa-miR-4763-3p	ATP11A	1704	-39.5	0.04189
hsa-miR-4763-3p	ATP11A	4045	-39.5	0.04189
hsa-miR-4763-3p	ATP11A	618	-39.0	0.05004
hsa-miR-4763-3p	ATP11A	2720	-38.9	0.05185
hsa-miR-4763-3p	ATP11A	2779	-38.9	0.05185

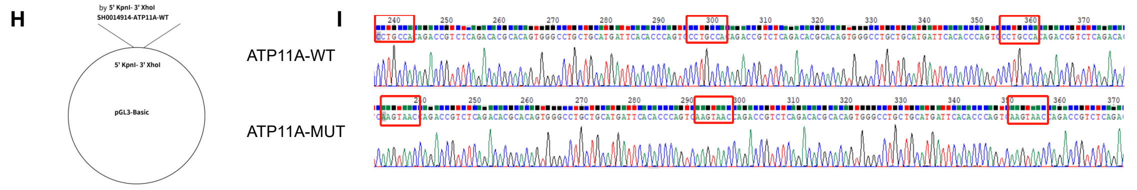

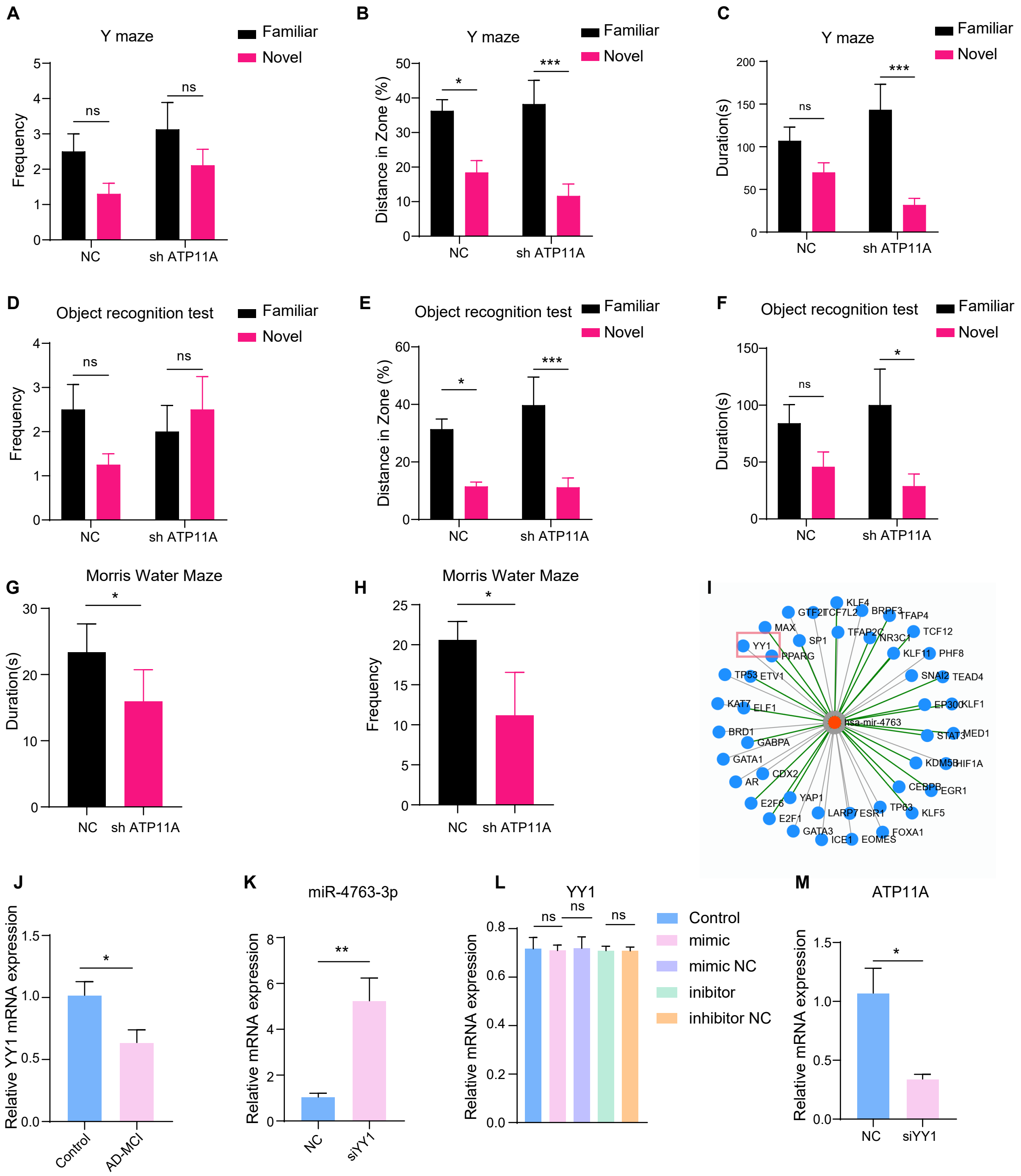
A
Y maze
Familiar
Novel
Frequency
ns
ns
NC
sh ATP11A
B
Y maze
Familiar
Novel
Distance in Zone (%)
*
***
NC
sh ATP11A
C
Y maze
Familiar
Novel
Duration(s)
ns
***
NC
sh ATP11A
D
Object recognition test
Familiar
Novel
Frequency
ns
ns
NC
sh ATP11A
E
Object recognition test
Familiar
Novel
Distance in Zone (%)
*
***
NC
sh ATP11A
F
Object recognition test
Familiar
Novel
Duration(s)
ns
*
NC
sh ATP11A
G
Morris Water Maze
Duration(s)
*
NC
sh ATP11A
H
Morris Water Maze
Frequency
*
NC
sh ATP11A
I
hsa-mir-4763
YY1
MAX
GTF2I
TCF7L2
KLF4
BRPF3
TFAP4
SP1
TFAP2C
NR3C1
TCF12
PPARG
KLF11
PHF8
TP53
ETV1
SNAI2
TEAD4
KAT7
ELF1
EP300
KLF1
BRD1
GABPA
STAT3
MED1
GATA1
CDX2
KDM5B
HIF1A
AR
CEBPB
EGR1
E2F6
YAP1
TP63
KLF5
E2F1
LARP7
ESR1
GATA3
ICE1
EOMES
FOXA1
J
Relative YY1 mRNA expression
*
Control
AD-MCI
K
miR-4763-3p
Relative mRNA expression
**
NC
siYY1
L
YY1
Relative mRNA expression
ns
ns
ns
Control
mimic
mimic NC
inibitor
inhibitor NC
M
ATP11A
Relative mRNA expression
*
NC
siYY1

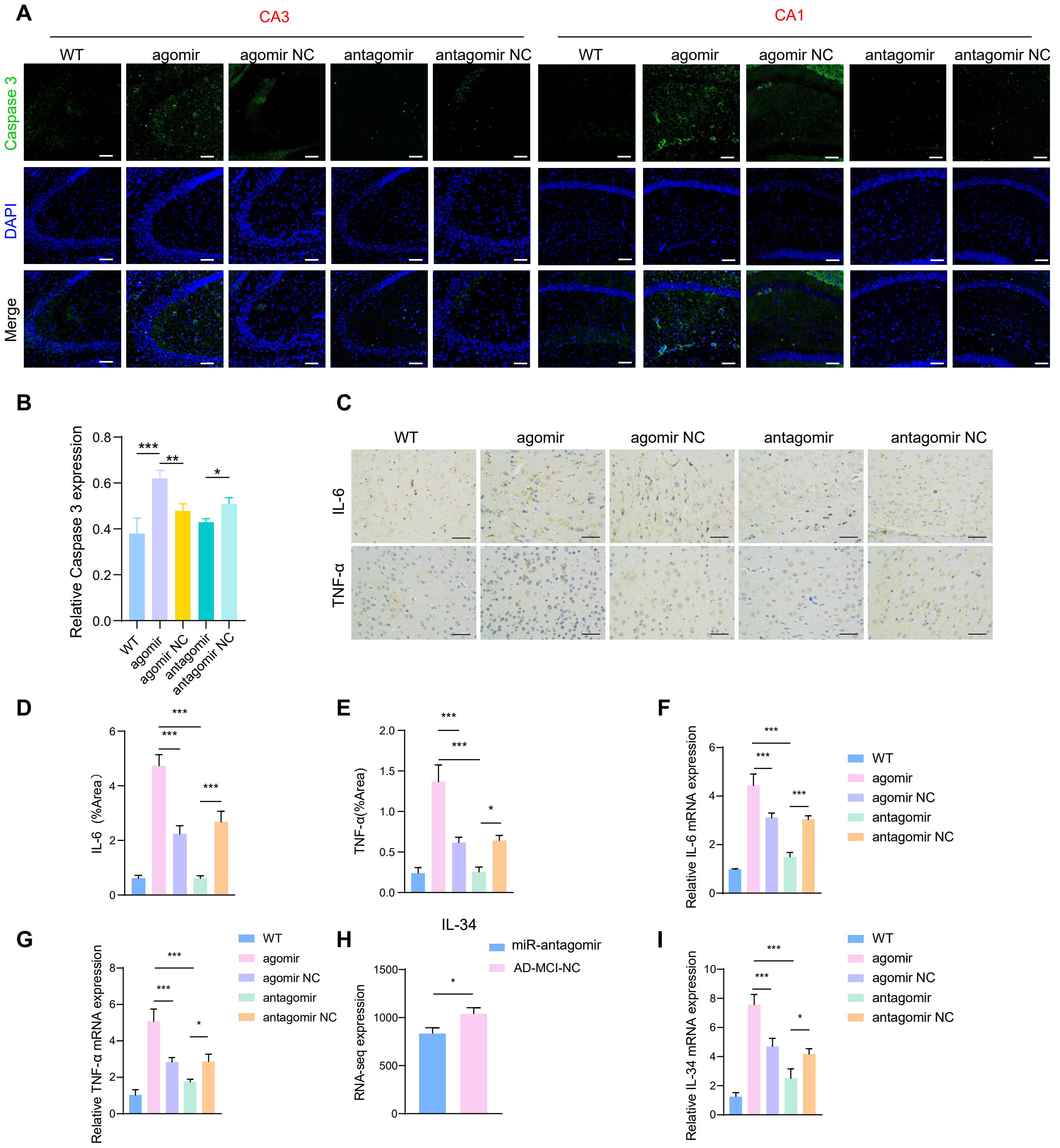
A
CA3
CA1
WT
agomir
agomir NC
antagomir
antagomir NC
Caspase 3
DAPI
Merge
B
Relative Caspase 3 expression
C
IL-6
TNF-α
D
IL-6 (%Area)
E
TNF-α(%Area)
F
Relative IL-6 mRNA expression
G
Relative TNF-α mRNA expression
H
IL-34
miR-antagomir
AD-MCI-NC
RNA-seq expression
I
Relative IL-34 mRNA expression

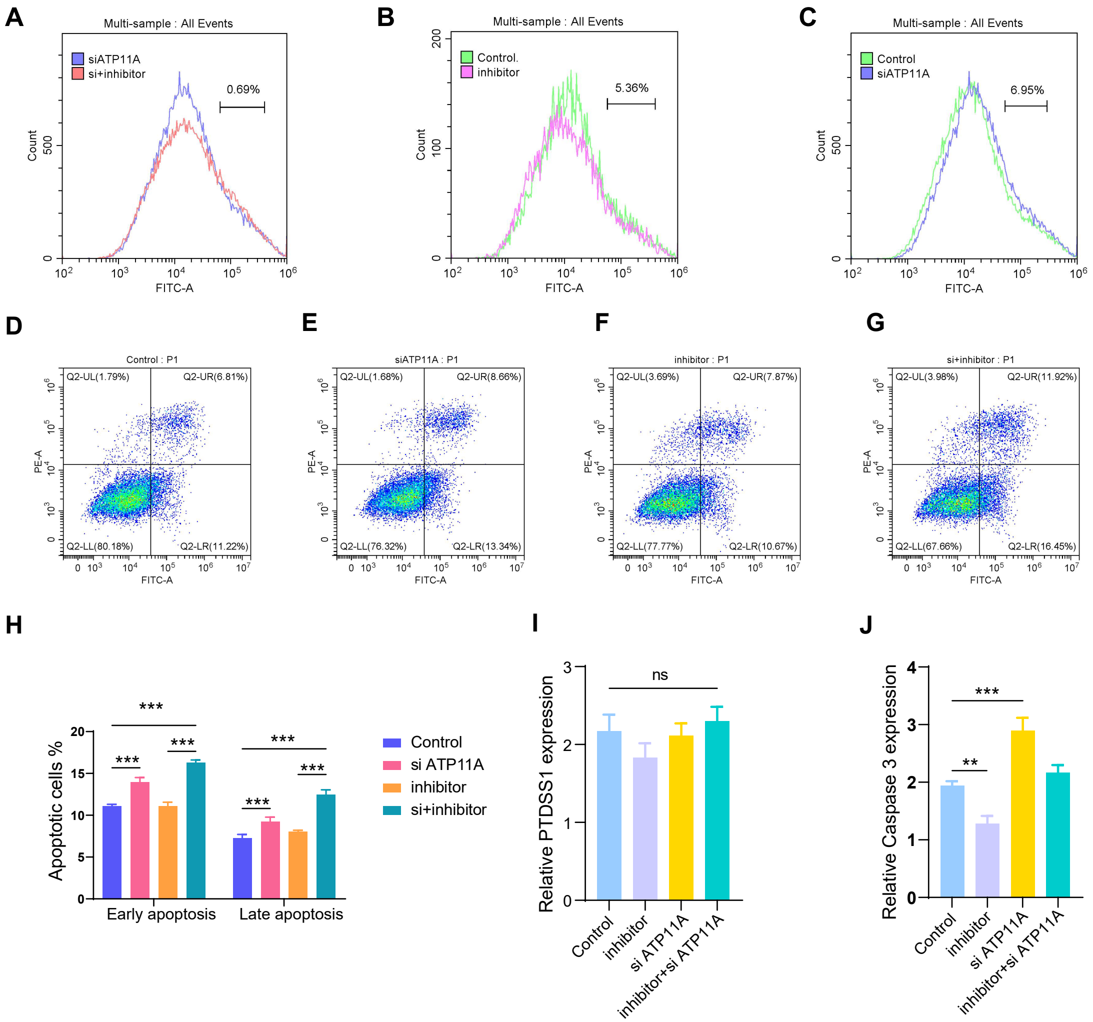
A
Multi-sample : All Events
siATP11A
si+inhibitor
0.69%
Count
FITC-A
B
Multi-sample : All Events
Control.
inhibitor
5.36%
Count
FITC-A
C
Multi-sample : All Events
Control
siATP11A
6.95%
Count
FITC-A
D
Control : P1
Q2-UL(1.79%)
Q2-UR(6.81%)
Q2-LL(80.18%)
Q2-LR(11.22%)
E
siATP11A : P1
Q2-UL(1.68%)
Q2-UR(8.66%)
Q2-LL(76.32%)
Q2-LR(13.34%)
F
inhibitor : P1
Q2-UL(3.69%)
Q2-UR(7.87%)
Q2-LL(77.77%)
Q2-LR(10.67%)
G
si+inhibitor : P1
Q2-UL(3.98%)
Q2-UR(11.92%)
Q2-LL(67.66%)
Q2-LR(16.45%)
PE-A
FITC-A
H
Apoptotic cells %
Early apoptosis
Late apoptosis
Control
si ATP11A
inhibitor
si+inhibitor
I
Relative PTDSS1 expression
ns
Control
inhibitor
si ATP11A
inhibitor+si ATP11A
J
Relative Caspase 3 expression
Control
inhibitor
si ATP11A
inhibitor+si ATP11A

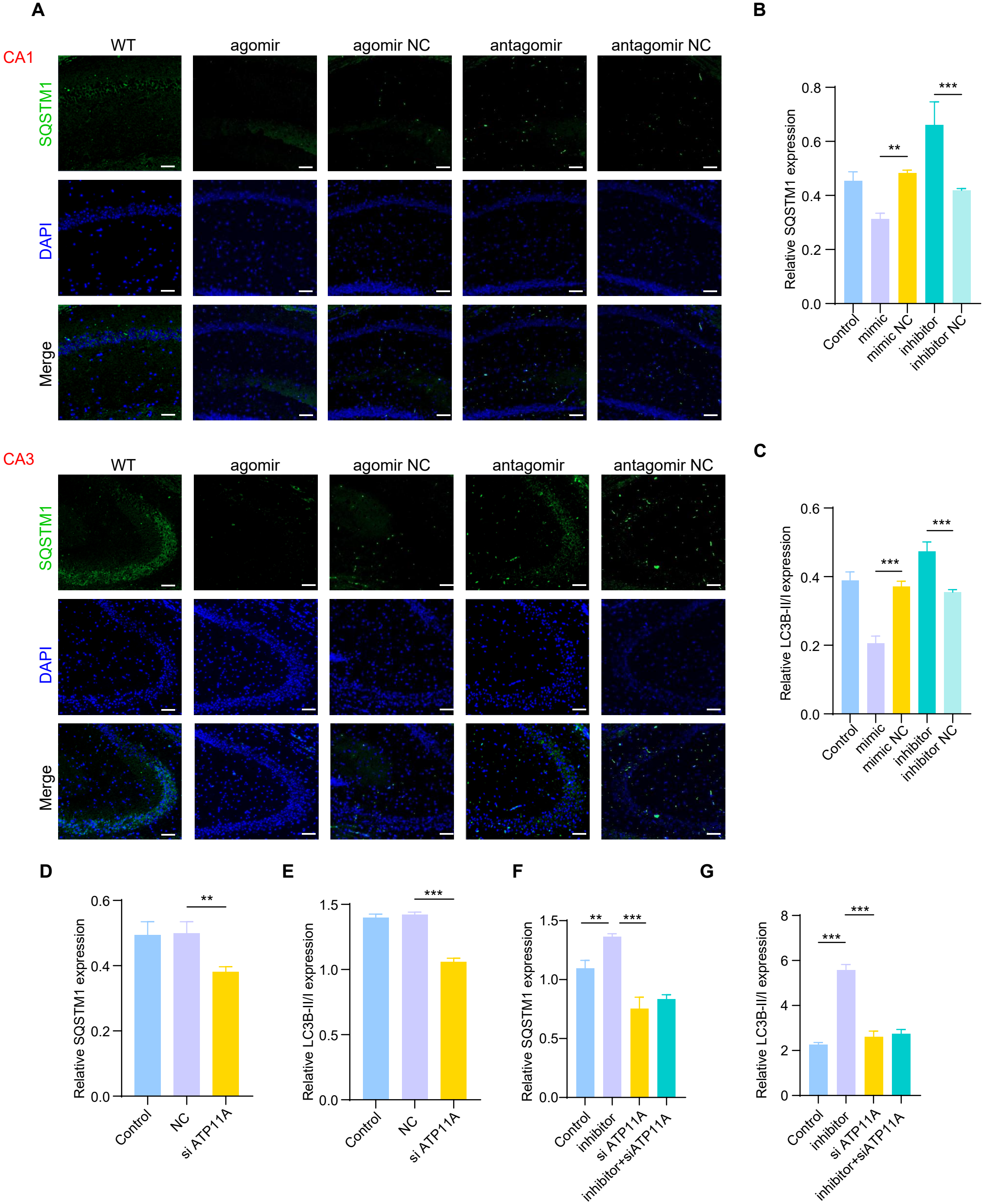

A
CA1
WT
agomir
agomir NC
antagomir
antagomir NC
SQSTM1
DAPI
Merge
CA3
B
Relative SQSTM1 expression
0.8
0.6
0.4
0.2
0.0
**
***
Control
mimic
mimic NC
inhibitor
inhibitor NC
C
Relative LC3B-II/I expression
D
Relative SQSTM1 expression
Control
NC
si ATP11A
E
Relative LC3B-II/I expression
1.5
1.0
0.5
F
si ATP11A
inhibitor+siATP11A
G
8
6
4
2
0

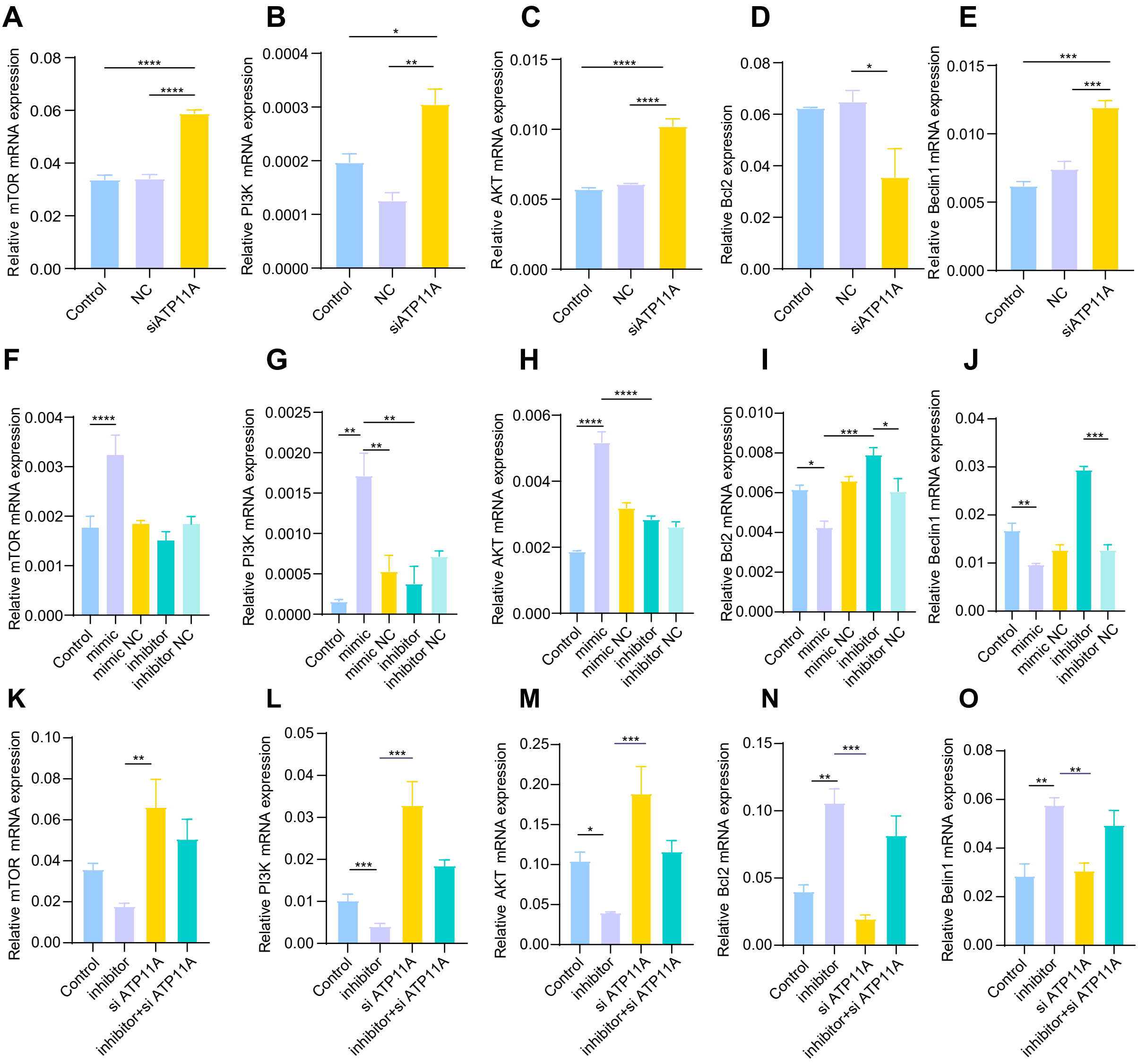
A
B
C
D
E
F
G
H
I
J
K
L
M
N
O
Relative mTOR mRNA expression
Relative PI3K mRNA expression
Relative AKT mRNA expression
Relative Bcl2 expression
Relative Beclin1 mRNA expression
Relative Bcl2 mRNA expression
Relative Belin1 mRNA expression
Control
NC
siATP11A
mimic
mimic NC
inhibitor
inhibitor NC
si ATP11A
inhibitor+si ATP11A

Figure S1 Inhibition of miR-4763-3p improves behavioral phenotyping in AD-MCI mice.

A: Volcano map of differentially expressed genes in serum non-coding RNA sequencing between AD patients and healthy controls, with red representing up-regulated genes in AD and blue representing down-regulated genes. $|log2FC|>$ 0.15 and $p < 0.05$.

B: The MWM test was performed to analyze long-term memory. The frequence of occupancy of the target quadrant were measured (n = 10 per group).

C: The object recognition test was conducted to assess the spatial memory capacity. The frequence of exploration of objects were recorded (n = 10 per group).

D: The Y maze test was conducted to evaluate the spatial memory capacity. The frequency of exploration of the novel and familiar arms were recorded (n = 10 per group).

E: The Barnes maze test was performed to assess changes in cognitive ability. The path lengh (cm) to the target hole and the path length were recorded (n = 10 per group).

F: The motor ability of object recognition test exploration of objects were recorded.

Data are expressed as the mean ± SEM of three independent experiments. **P < 0.05; **P < 0.01; ***P < 0.001; ****P < 0.0001.*

Figure S2 Silencing of miR-4763-3p rescues the synaptic disorder in AD mice.

A: Golgi staining shows dendritic spine morphology injected with antagomir NC, antagomir, agomir, agomir NC. Scale bar, 200 or 50μm.

B, C: qRT-PCR analysis was performed to detect GLUR1 and GLUR2 in hippocampal homogenates from five different groups (n = 4).

D: The differential gene expression heatmap identified by RNA-seq for mice injected with the miR-4763-3p antagomir was significantly different from that for mice injected with the antagomir NC (AD-MCI-NC) (n = 3 per group).

E: The volcano plot shows a log2-fold change on the x-axis and statistical

significance on the y-axis, with genes significantly different in abundance between mice injected with AD-MCI-NC and those injected with the miR-4763-3p antagomir. The names of some differentially expressed genes are shown.

F: Volcano maps show differences in gene expression in hippocampal tissue after death between healthy controls and Alzheimer's patients.

G: GO enrichment analysis of differentially expressed genes. Data from GEO database (GSE173955).

Data are expressed as the mean ± SEM of three independent experiments. **P < 0.05; **P < 0.01; ***P < 0.001; ****P < 0.0001.*

Figure S3 MiR-4763-3p targets ATP11A.

A, B: Differences in the expression of Atp11a between WT (C57BL/6) and AD (AppNL-G-F) mice at different ages and in different brain regions. Data were taken from the Alzmap database.

C: RT-qPCR of the transfection efficiency of siATP11A.

D: RT-qPCR analysis of ATP11A expression in SH-SY5Y cells transfected with mimic NC, mimic, inhibitor, inhibitor NC and control.

E: Immunoblotting was evaluated the expression level of ATP11A in mouse hippocampal tissue from the antagomir NC, antagomir, agomir NC, and agomir groups.

F: Prediction of the binding sequence for miR-4763-3p and ATP11A using the RNAhybrid database.

G: Binding free energy of miR-4763-3p and ATP11A.

H, I: Plasmids were constructed according to the binding site between miR-4763-3p and wild-type ATP11A (ATP11A-WT); miR-4763-3p and mutant ATP11A (ATP11A-MUT).

Data are expressed as the mean ± SEM of three independent experiments. *P < 0.05; **P < 0.01; ***P < 0.001; ****P < 0.0001.

Figure S4 The mechanism of miR-4763-3p/ATP11A/YY1 axis on cognitive

decline.

A-C: The Y maze test was conducted to assess spatial memory capacity. Frequency, distance in zone and duration of exploration of novel and familiar arms were recorded (n = 10 per group).

D-F: The object recognition tests were conducted to assess spatial memory capacity. The duration, distance in zone and frequency of exploration of novel object was recorded (n = 10 per group).

G, H: The morris water maze test was performed to analyze long-term memory. The duration and frequency of occupancy of the platform area was measured. The long-term memory performance of shATP11A mice was significantly reduced compared to NC mice. (n =10 per group).

I: Transcription factor network analysis for miR-4763-3p.

J: RT-qPCR was performed to detect YY1 expression levels in hippocampal tissues of Control and AD-MCI mice (n=4).

K: RT-qPCR was performed to detect the expression level of miR-4763-3p in SH-SY5Y transfected with NC or siYY1.

L: RT-qPCR was performed to detect the expression level of YY1 in SH-SY5Y transfected with mimic NC, mimic, inhibitor, inhibitor NC and control.

M: RT-qPCR was performed to detect the expression level of ATP11A in SH-SY5Y transfected with NC or siYY1.

Data are expressed as the mean ± SEM of three independent experiments. **$P < 0.05$; **$P < 0.01$; ***$P < 0.001$; ****$P < 0.0001$.*

Figure S5 MiR-4763-3p antagomir targets ATP11A to rescue early apoptosis.

A, B: Immunofluorescence staining of caspase 3 (green) in the CA1 and CA3 hippocampal regions of the mice brain from the five groups. Nuclei were stained with DAPI (blue). Scale bar, 100μm. The histograms show the relative expression of caspase 3.

C: Immunohistochemistry (IHC) staining for IL-6 and TNF-α in the mice brain from the five groups, scar bar，50 μ m.

D, E: IL-6 and TNF-α positive areas were quantified after IHC assays (n=3).

F, G: RT-qPCR was performed to detect the expression level of IL-6 and TNF-α in brain tissue.

H, I: IL-34 expression in miR-antagomir and AD-MCI-NC sequencing data(H); IL-34 expression level in five groups was detected by qPCR(I).

Data are expressed as the mean ± SEM of three independent experiments. *$P < 0.05$; **$P < 0.01$; ***$P < 0.001$.

Figure S6 MiR-4763-3p antagomir targets ATP11A to rescue apoptosis in neurons

A-C: Flow cytometry analysis of PS exposure in SH-SY5Y cells transfected with the miR-4763-3p inhibitor and siATP11A.

D-H: Flow cytometry analysis of the apoptotic rate of SH-SY5Y cells transfected with the miR-4763-3p inhibitor or siATP11A. The histogram shows the percentage of early and late apoptotic cells.

I, J: The histograms show the relative protein expression of PTDSS1 and caspase 3.

Data are expressed as the mean ± SEM of three independent experiments. *$P < 0.05$; **$P < 0.01$; ***$P < 0.001$.

Figure S7 MiR-4763-3p antagomir/ATP11A increases autophagic flux in neurons.

A: IF staining of SQSTM1 (green) in the hippocampal region of mice brain from the antagomir NC, antagomir, agomir NC, and agomir groups. Nuclei were stained with DAPI (blue). Scale bar, 50μm.

B-G: Western blot analysis of autophagy flux markers in SH-SY5Y cell transfected mimic, mimic NC, inhibitor, inhibitor NC and control groups (B, C); NC, siATP11A (D, E) and inhibitor, siATP11A and inhibitor co-transfected with siATP11A (F, G).

Data are expressed as the mean ± SEM of three independent experiments. *$P < 0.05$; **$P < 0.01$; ***$P < 0.001$; ****$P < 0.0001$.

Figure S8 miR-4763-3p antagomir/ATP11A inhibits the PI3K/AKT/mTOR signaling pathway to mediate crosstalk between autophagy and apoptosis

RT-qPCR analysis of mTOR, PI3K, AKT, Beclin1, and Bcl2 mRNA expression. GAPDH served as an internal reference gene. Data are expressed as the mean ± SD

of three independent experiments. $^{*}P < 0.05$; $^{**}P < 0.01$; $^{***}P < 0.001$; $^{****}P < 0.0001$.

Table S1: List of PCR and qPCR primers used in this study

Primers for qPCR (Name ID-human)	
U6-F	TGGAACGATACAGAGAAGATTAGCA
U6-R	TATGGAACGCTTCACGAATTTGC
GAPDH-F	GGAGCGAGATCCCTCCAAAAT
GAPDH-R	GGCTGTTGTCATACTTCTCATGG
ATP11A-F	CAAACAGGGTTATGAAGACTGGC
ATP11A-R	TGCCGTGCTGAATGAAATGAA
Caspase3-F	GAAATTGTGGAATTGATGCGTGA
Caspase3-R	CTACAACGATCCCCTCTGAAAAA
PTDSS1-F	GCAAGTGGAGGACATCACCAT
PTDSS1-R	TCATCCCTGGTAAAGGCGAAG
GLUR1-F	CGAGCTTTCCCGTTGATACAT
GLUR1-R	CATTCAGATGAGACCCGACCT
GLUR2-F	TCTGCCACTTGTAATGGTCAATG
GLUR2-R	GGTATGCAAACTTGTCCCATTGA
YY1-F	ACGGCTTCGAGGATCAGATTC
YY1-R	TGACCAGCGTTTGTTCAATGT
BCL2-F	GGTGGGGTCATGTGTGTGG
BCL2-R	CGGTTCAGGTACTCAGTCATCC
LC3B-F	AAGGCGCTTACAGCTCAATG
LC3B-R	CTGGGAGGCATAGACCATGT
SQSTM1-F	GACTACGACTTGTGTAGCGTC
SQSTM1-R	AGTGTCCGTGTTTCACCTTCC
TNF-α-F	GACGTGGAACTGGCAGAAGAG
TNF-α-R	TTGGTGGTTTGTGAGTGTGAG
IL-6-F	TAGTCCTTCCTACCCCAATTTCC
IL-6-R	TTGGTCCTTAGCCACTCCTTC
MTOR-F	TCCGAGAGATGAGTCAAGAGG
MTOR-R	CACCTTCCACTCCTATGAGGC
AKT-F	AGCGACGTGGCTATTGTGAAG
AKT-R	GCCATCATTCTTGAGGAGGAAGT
Beclin1-F	GGTGTCTCTCGCAGATTCATC
Beclin1-R	TCAGTCTTCGGCTGAGGTTCT
PI3K-F	CCACGACCATCATCAGGTGAA
PI3K-R	CCTCACGGAGGCATTCTAAAGT

Table S2: List of PCR and qPCR primers used in this study

Primers for qPCR (Name ID-mouse)	
**PI3K-F**	**ACACCACGGTTTGGACTATGG**
**PI3K-R**	**GGCTACAGTAGTGGGCTTGG**
**AKT-F**	**ATGAACGACGTAGCCATTGTG**
**AKT-R**	**TTGTAGCCAATAAAGGTGCCAT**
**MTOR-F**	**CACCAGAATTGGCAGATTTGC**
**MTOR-R**	**CTTGGACGCCATTTCCATGAC**
**GAPDH-F**	**AGGTCGGTGTGAACGGATTTG**
**GAPDH-R**	**GGGGTCGTTGATGGCAACA**
**ATP11A-F**	**GCTTACTACATTGGCCCAGATT**
**ATP11A-R**	**AGGCTGGTACATGGTCTTGAA**
**Caspase3-F**	**ATGGAGAACAACAAAACCTCAGT**
**Caspase3-R**	**TTGCTCCCATGTATGGTCTTTAC**
**PTDSS1-F**	**GCAGGACTCTGAGCAAGGATG**
**PTDSS1-R**	**GGCGAAGTACATGAGGCTGAT**
**GLUR1-F**	**TCCCCAACAATATCCAGATAGGG**
**GLUR1-R**	**AAGCCGCATGTTCCTGTGATT**
**GLUR2-F**	**TTCTCCTGTTTTATGGGGACTGA**
**GLUR2-R**	**CACTCTCGATGCCATATACGTTG**
**YY1-F**	**CAGTGGTTGAAGAGCAGATCAT**
**YY1-R**	**AGGGAGTTTCTTGCCTGTCAT**
**BCL2-F**	**GTCGCTACCGTCGTGACTTC**
**BCL2-R**	**CAGACATGCACCTACCCAGC**
**LC3B-F**	**TTATAGAGCGATACAAGGGGGAG**
**LC3B-R**	**CGCCGTCTGATTATCTTGATGAG**
**SQSTM1-F**	**AGGATGGGGACTTGGTTGC**
**SQSTM1-R**	**TCACAGATCACATTGGGGTGC**
**TNF-α-F**	**CCCTCACACTCAGATCATCTTCT**
**TNF-α-R**	**GCTACGACGTGGGCTACAG**
**IL-6-F**	**TAGTCCTTCCTACCCCAATTTCC**
**IL-6-R**	**TTGGTCCTTAGCCACTCCTTC**
**Beclin1-F**	**ATGGAGGGGTCTAAGGCGTC**
**Beclin1-R**	**TCCTCTCCTGAGTTAGCCTCT**

Table S3: List of PCR and qPCR primers used in this study

Primers for CHIP-qPCR (Name ID)	
ATP11A-0kb-F	GCTGCTGCATTGTCAACCAA
ATP11A-0kb-R	GGGTGAGACATGTTGTCGGT
ATP11A -(+0.5kb)-F	CTGGGACCCTGGCTGAAAC
ATP11A -(+0.5kb)-R	CTTCTCTCTCCCCGTCGGTC
ATP11A -(+4kb)-F	TCCAGCTGACTTCGGTGAAC
ATP11A -(+4kb)-R	CTGGAGGGGAAATGGCTCAG
ATP11A -(+3kb)-F	TGTGGGTCCCTAGACACCTT
ATP11A -(+3kb)-R	TGTCCCCCTCCCAAGATGAA
ATP11A -(+5kb)-F	ACATAGCTGAGTCCCTGGGT
ATP11A -(+5kb)-R	TCCACACCATGCTCAGTGTC
ATP11A -(-3kb)-F	AGGTTAGAGAATGGCCTGCG
ATP11A -(-3kb)-R	CCTAGGTGACCATCTCCCCA
ATP11A -(-5kb)-F	GAAACGGAGCCAATGATCGC
ATP11A -(-5kb)-R	CACACCCATCCTGAGACACC

Table S4: The sequences of siRNAs

(Name ID)	Target sequence
SiATP11A-#1-F	GCAUAGGUGUCAUCGGCAATT
SiATP11A-#1-R	UUGCCGAUGACACCUAUGCTT
SiATP11A -#2-F	GACGUUUGGAACGCUGGUAUU
SiATP11A -#2-R	AAUACCAGCGUUCCAAACGUC
SiATP11A -#3-F	CCAGAGGAUGUACUACGUGUU
SiATP11A -#3-R	AACACGUAGUACAUCCUCUGG

Table S5: miR-4763-3p agomir/antagomir sequence information list

(Name ID)	Target sequence
miR-4763-3p antagomir	CCCGCCCAGCACCAGCCCCUGCCU
miR-4763-3p agomir	AGGCAGGGGCUGGUGCUGGGCGGG

Table S6: List of Antibodies used in this study

Antibody	Company	Cat. No.	Species
p-PI3K	Cell Signaling Technology	4228T	Rabbit
p-PI3K	Abways	CY6428	Rabbit
PI3K	Proteintech	67644-1-Ig	Mouse
p-AKT	Abways	CY6569	Rabbit
AKT	Abways	CY5561	Rabbit
mTOR	Abways	CY8744	Rabbit
p-mTOR	Abways	CY5996	Rabbit
Bcl2	ABclonal	A19693	Rabbit
Beclin1	ABclonal	A21191	Rabbit
Caspase3	ABclonal	A0214	Rabbit
YY1	Cell Signaling Technology	46395S	Rabbit
IL-6	ABclonal	A21264	Rabbit
TNF-α	ABclonal	A11534	Rabbit
NeuN	Abcam	ab177487	Rabbit
Iba1	Abcam	ab178846	Rabbit
Annexin V	Abways	CY6721	Rabbit
SQSTM1	ABclonal	A19700	Rabbit
β-Amyloid(1-42)	ABclonal	A24422	Rabbit
beta Tubulin	Bioss	bsm-33034M	Mouse
ATP11A	Invitrogen	PA5-20995	Rabbit
beta Actin	Abways	AB2001	Mouse
IgG	ABclonal	AC011	Mouse
GAPDH	Abways	AB0036	Rabbit
Vinculin	Abways	CY5164	Rabbit
